# Comprehensive comparison of different parts of *Paeonia ostii*, a food-medicine plant, based on untargeted metabolomics, quantitative analysis, and bioactivity analysis

**DOI:** 10.3389/fpls.2023.1243724

**Published:** 2023-08-29

**Authors:** Yaping Zheng, Pei Li, Jie Shen, Kailin Yang, Xinyan Wu, Yue Wang, Yu-he Yuan, Peigen Xiao, Chunnian He

**Affiliations:** ^1^ Key Laboratory of Bioactive Substances and Resources Utilization of Chinese Herbal Medicine, Ministry of Education, Institute of Medicinal Plant Development, Chinese Academy of Medical Science, Peking Union Medical College, Beijing, China; ^2^ School of Laboratory Medicine, Key Laboratory of Clinical Laboratory Diagnostics in Universities of Shandong, Weifang Medical University, Weifang, Shandong, China; ^3^ Institute of Materia Medica, Chinese Academy of Medical Sciences and Peking Union Medical College, Beijing, China

**Keywords:** *Paeonia ostii*, quantitative analysis, metabolomics, different parts, bioactivity

## Abstract

**Introduction:**

*Paeonia ostii* T. Hong & J.X. Zhang (s.s.) (Chinese name, Fengdan) is a widely cultivated food-medicine plant in China, in which root bark, seed kernels, and flowers are utilized for their medicinal and edible values. However, other parts of the plant are not used efficiently, in part due to a poor understanding of their chemical composition and potential biological activity.

**Methods:**

Untargeted ultra-performance liquid chromatography–quadrupole time of flight–mass spectrometry (UPLC-Q-TOF-MS) metabolomics was applied to characterize the metabolic profiles of 10 different parts of *P. ostii*.

**Results and discussion:**

A total of 160 metabolites were alternatively identified definitely or tentatively, which were significantly different in various plant parts by multivariate statistical analysis. Quantitative analysis showed that underutilized plant parts also contain many active ingredients. Compared with the medicinal part of root bark, the root core part still contains a higher content of paeoniflorin (17.60 ± 0.06 mg/g) and PGG (15.50 ± 2.00 mg/g). Petals, as an edible part, contain high levels of quercitrin, and stamens have higher methyl gallate and PGG. Unexpectedly, the ovary has the highest content of methyl gallate and rather high levels of PGG (38.14 ± 1.27 mg/g), and it also contains surprisingly high concentrations of floralalbiflorin I. Paeoniflorin (38.68 ± 0.76 mg/g) is the most abundant in leaves, and the content is even higher than in the root bark. Branches are also rich in a variety of catechin derivatives and active ingredients such as hydrolyzable tannins. Seed kernels also contain fairly high levels of paeoniflorin and albiflorin. Fruit shells still contain a variety of components, although not at high levels. Seed coats, as by-products removed from peony seeds before oil extraction, have high contents of stilbenes, such as *trans*-gnetin H and suffruticosol B, showing significant potential for exploitation. Except for the seed kernels, extracts obtained from other parts exhibited good antioxidant activity in DPPH, ABTS, and ferric ion reducing antioxidant power (FRAP) assays (0.09–1.52 mmol TE/g). Five compounds (gallic acid, PGG, *trans*-resveratrol, kaempferol, and quercitrin) were important ingredients that contributed to their antioxidant activities. Furthermore, *P. ostii* seed cakes were first reported to possess agonistic activity toward CB1/CB2 receptors. This study provides a scientific basis for the further development and utilization of *P. ostii* plant resources.

## 1 Introduction

*Paeonia ostii* T. Hong & J.X. Zhang (s.s.), commonly known as Fengdan in Chinese, is a woody shrub belonging to the *Paeonia*ceae, and its wild species has been endangered (Hong, 2010; Hong, 2011). In ancient China, people cultivated this crop for its traditional medicinal benefits, particularly the roots and flowers. The dried root bark of *P. ostii* is widely used as a primary source of the traditional medicine “*Cortex Moutan*” (CM), valued for its heat-clearing and blood-cooling effects, as well as its ability to promote blood circulation and alleviate blood stasis. CM holds important applications in various herbal medicinal systems across Asia for the treatment of inflammatory, cardiovascular, and gynecological diseases (Li et al., 2021). Additionally, the *Compendium of Materia Medica* documents the utilization of *P. ostii* flowers as a traditional medicinal herb renowned for its heat-clearing and detoxification properties (Yang et al., 2011). Pharmacological studies have further revealed that the flowers of *P. ostii* exhibit antioxidant and anti-inflammatory activities (Zhang H, et al., 2017). Furthermore, *P. ostii* flowers also possess edible values, and the history of people eating *P. ostii* flowers can be traced back to the Song Dynasty (960 AD). *P. ostii* has been recognized as a new food resource due to its nutritional value (Yang et al., 2011). In modern times, people commonly consume *P. ostii* flowers in various forms such as tea, cakes, beverages, and wine.

In the past, *P. ostii* was primarily cultivated for its medicinal properties. However, in recent times, it has gained popularity as an oil crop. Much research reported that peony seed oil extracted from *P. ostii* was characterized by high contents of unsaturated fatty acid (UFA) (>90%) and a high ratio of α-linolenic acid (approximately 45%) (Li et al., 2015). Due to its exceptional seed quality and high seed oil yield, *P. ostii* has been considered one of the significant sources of new woody oil grain crops. In 2011, the Ministry of Health of China approved peony seed oil as a new resource for food, gaining a significant surge in attention. As a result, the cultivation area of oil peony is growing rapidly. It is estimated that the oil peony planting area in China was approximately 20,000 hm^2^ in 2013 and has expanded to nearly 666,666 hm^2^ in 2020 (Mao et al., 2017; Yang et al., 2017; Zhou et al., 2020). This substantial increase in cultivation area also reflects the growing attention and interest in peony seed oil.

In the last few decades, mass spectrometry-based untargeted metabolomics has been widely used to comprehensively analyze the chemical composition in plant extracts (Vinaixa et al., 2016; Rocchetti et al., 2019; Yoo et al., 2021). By detecting as many features as possible, untargeted metabolomics combined with multivariate statistics allows for holistic analysis when comparing complex plant samples. To the best of our knowledge, there has been no comprehensive comparative study on the metabolic profiles of the whole plant of *P. ostii*. In addition, previous research has focused on a few commonly used parts of *P. ostii*, such as roots, leaves, seed kernels, and flowers, in order to investigate their pharmacological activities, especially concerning antioxidant activity, which are closely linked to various diseases. However, there are few biological studies on other neglected parts of *P. ostii*.

In recent years, there have been multiple studies conducted on the phytochemical and biological properties of *P. ostii*. These studies have isolated numerous bioactive components, such as monoterpene glycosides, flavonoids, tannins, triterpenes, and phenols, which have demonstrated various beneficial activities including antioxidant, anti-inflammatory, anti-tumor, cardiovascular-protective, and neuroprotective effects (Zhao and Wu, 2019). Root bark, flowers, and seed kernels are the plant parts that receive the most attention in current research (Wang et al., 2017a; Liu et al., 2019; Tian et al., 2020; Zhang H, et al., 2017). However, other parts such as root core, branches, leaves, seed coats, and fruit shells are usually discarded as by-products due to their perceived lack of utility. Considering the substantial biomass of these plant parts, the comprehensive utilization of *P. ostii* has garnered the attention of the authorities including producers and environmental protection. However, the limited understanding of the phytochemical composition and potential biological activities of these plant parts has hindered their exploitation and utilization.

In this study, we compared the phytochemical characteristics of 10 different parts of *P. ostii* based on untargeted ultra-performance liquid chromatography–quadrupole time of flight–mass spectrometry (UPLC-Q-TOF-MS) metabolomics and content determination of important components. Moreover, we evaluated the antioxidant and cannabinoid receptor CB1/CB2 agonistic activities in these 10 plant parts and explored the correlation between metabolites and the activities of crude extracts. The present study provides a scientific basis for the comprehensive utilization of different parts of *P. ostii* as medicinal and edible resources, especially the plant parts that have been neglected in the long term.

## 2 Materials and methods

### 2.1 Plant materials, chemicals, and reagents

In 2017, samples of 10 different parts of *P. ostii* were collected in different seasons in the Haidian District, Beijing City. The leaves, petals, stamens, and ovary were collected in early May, while the branches, seed coats, seed kernels, and fruit shells were collected in mid-September. Additionally, root bark and root core were collected at the end of October. Three samples were collected in parallel from each part of *P. ostii*. The voucher specimens were identified and verified by Professor Chunnian He from the Institute of Medicinal Plant Development (IMPLAD) and then deposited in the Pharmacophylogeny Centre of IMPALD in Beijing, China. Detailed information on samples can be found in **Table S1**.

As a crop with both medicinal and edible values, the different plant parts of *P. ostii* were collected separately at the appropriate growing season in the current study. The root core is a by-product of the root bark as a medicinal part being stripped and processed separately. Branches were usually pruned in autumn to enhance plant growth. Early summer is the peak growing season of *P. ostii* leaves, so they are collected at this time. Flowers of *P. ostii* were harvested during the blooming seasons, and the petals and stamens were processed as raw materials for food and cosmetics. In certain cases, *P. ostii* was cultivated as a medicinal plant, and people harvested its buds in early spring to facilitate enhanced nutrient absorption in the roots. The fruits developed from the ovary are typically harvested from July to September and used as a raw material for seed oil. As a matter of fact, *P. ostii* is cultivated on a large scale as a promising oil crop, resulting in significant production of fruit shells and seed coats. However, these by-products are not effectively utilized. Currently, people tend to discard the fruit shells and seed coats and only extract edible oil from the seed kernels, which are rich in unsaturated fatty acids.

Authentic standard compounds were applied to compare the retention time, MS1, and MS2 data with identified metabolites, including 28 compounds. Nineteen of them (gallic acid, methyl gallate, oxypaeoniflorin, catechin, paeonolide, apiopaeonoside, albiflorin, paeoniflorin, galloylpaeoniflorin, PGG, quercitrin, benzoic acid, mudanpioside C, benzoyl oxypaeoniflorin, quercetin, kaempferol, paeonol, *trans*-resveratrol, and benzoylpaeoniflorin) were purchased from Chengdu Puruifa Technology Development Co., Ltd. The other nine compounds (suffruticosol A, suffruticosol B, suffruticosol C, *cis*-viniferin, *trans*-viniferin, *trans*-gnetin H, *cis*-gnetin H, *cis*-suffruticosol D, and *trans*-suffruticosol D) were isolated from *P. ostii* in our laboratory and achieved a purity > 95% after thin-layer chromatography (TLC) and high-performance liquid chromatography (HPLC)-UV detection (Liu et al., 2020). For UPLC-Q-TOF-MS and UPLC–diode array detection (DAD) analysis, chromatographic grade methanol, acetonitrile, and formic acid were purchased from Sigma-Aldrich (St. Louis, MO, USA). The analytical grade methanol used for sample extraction was purchased from Beijing Chemical Reagent Company (Beijing, China), and all solutions were prepared with ultrapure water (Milli-Q, Millipore Company, Billerica, MA, USA). Diphenyl-1-picrylhydrazyl (DPPH) was supplied by Sigma-Aldrich. The 2,2′-azino-bis(3-ethylbenzothiazoline-6-sulfonic acid) (ABTS) and ferric ion reducing antioxidant power (FRAP) test kits were purchased from Shanghai Biyuntian Biotechnology Co., Ltd. (Shanghai, China).

### 2.2 Sample solution preparation

Plant samples were first dried, crushed, and sieved through a no. 60 mesh. Then, the dried sample powder (1.0 g) was ultrasonically extracted with 70% methanol–water mixed solvent (100 mL) for 30 min. Obtained supernatant after centrifugation was filtered and stored at 4°C for subsequent UPLC-Q-TOF-MS and UPLC-DAD analyses.

To evaluate the antioxidant and agonistic activities of cannabinoid receptor CB1/CB2, the powdered samples (0.5 g) were ultrasonically extracted three times (20 W, 40 kHz) with 50 mL of a mixed solvent of methanol and water (70:30 v/v), each for 30 min. The solutions were collected and filtered with qualitative filter paper and concentrated using a rotary evaporator (N-1000SWD, Eyela, Tokyo, Japan). Afterward, the crude extraction was freeze-dried in a freeze dryer under the following conditions: the temperature of the cold trap was −50°C, and the pressure was 0.3 mbar (Lyovapor L-300, BUCHI, Flawil, Switzerland). Finally, the obtained crude extraction powder was hermetically sealed and stored in opaque glass bottles at 4°C until further use. For the assessment of antioxidant and CB1/CB2 receptor agonistic activities, 10 mg of each sample extraction powder was dissolved in 5 mL by 70% methanol–water mixed solvent and 1 mL by dimethyl sulfoxide (DMSO).

### 2.3 Chromatographic and mass spectrometry conditions

The chromatographic and mass spectrometry conditions of UPLC-Q-TOF-MS analysis were the same as reported in our lab (Wang et al., 2019). Briefly, a Waters ACQUITY-UPLC CLASS (Waters Corp., Milford, MA, USA) system equipped with Agilent ZORBAX Eclipse Plus C18 column (1.8 μm × 2.1 mm × 100 mm) was used, the column temperature was 45°C, the flow rate was 0.3 mL/min, and the injection volume was 1 μL. The mobile phase consisted of (solvent A) 0.1% formic acid–water and (solvent B) acetonitrile, and the gradient was as follows: 0–2 min, 6%–15% B; 2–3 min, 15–16% B; 3–6 min, 16–24% B; 6–10 min, 24–33% B; 10–13 min, 33–43% B; 13–15.5 min, 43–95% B; 15.5–17.5 min, 95% B. A Waters Q-TOF-MS system (SYNAPT G2-Si, Waters) equipped with an electrospray source was used to obtain MS data in negative ion mode, and the mass scan range was 50–1,500 Da. The data processing and compound identification of untargeted metabolomics were performed in Progenesis QI 2.3 software (Waters, Milford, MA, USA), and an in-house database used in this study is consistent with that reported in the previous literature (Wang et al., 2019). The in-house database including 464 compounds previously isolated from genus *Paeonia* was imported into Progenesis QI for compound identification, including their name, chemical formula, formula weight, SMILES code, and chemical classes. The RAW format acquisition data and SDF format in-house database were imported into Progenesis QI software for peak extraction, peak alignment, normalization, and compound identification. The pre-processing resulted in a metabolic features matrix containing 3,840 metabolic features followed by filtering with a coefficient of variation (CV) < 30%, and their retention time, m/z values, and peak abundance were obtained. The feature identification results were also exported after manual confirmation.

The UPLC-DAD quantification methods were employed to measure the content of several important biomarker components. Stilbenes and other constituents (monoterpene glycosides, flavonoids, phenolic acids, etc.) were determined by two chromatographic conditions, separately. The chromatographic conditions of 10 stilbenes (suffruticosol A–C, *trans*-resveratrol, *cis*-*ϵ*-viniferin, *trans*-*ϵ*-viniferin, *cis*-suffruticosol D, *cis*-gnetin H, *trans*-suffruticosol D, and *trans*-gnetin H) were the same as those of the previous methods in our laboratory (He et al., 2016). Similarly, the chromatographic conditions for 18 other types of components (gallic acid, methyl gallate, oxypaeoniflorin, catechin, paeonolide, apiopaeonoside, albiflorin, paeoniflorin, benzoic acid, galloylpaeoniflorin, 1,2,3,4,6-penta-*O*-galloyl-*β*-d-glucose (PGG), quercitrin, mudanpioside C, benzoyl oxypaeoniflorin, quercetin, kaempferol, benzoylpaeoniflorin, and paeonol) are also the same as reported in the lab (Wang et al., 2017b).

### 2.4 Antioxidant activity assays

The DPPH radical scavenging antioxidant assays of plant crude extraction from 10 different parts of *P. ostii* were performed regarding the methods reported in the literature (Kolak et al., 2006; Bothon et al., 2013). Sample solution or Trolox solution (at six different concentrations) or blank methanol solution with a volume of 40 μL was added to 160 μL of 0.2 mM DPPH solution. After incubation for 30 min at 37°C, the absorbance was measured at 517 nm. Blank methanol solution and Trolox were used as blank control and positive control, respectively. The ABTS and FRAP assay kits were also used following the method outlined by Bi et al. (2016) to assess their ability to scavenge free radicals. The following formula was used to calculate the inhibition rate for three different assay systems: radical-scavenging activity (% inhibition) = (1 − Abs_sample_/Abs_control_) × 100%, in which Abs_control_ and Abs_sample_ refer to the absorbance of the blank solution and sample solution, respectively. Each test was repeated three times, and the results were expressed as Trolox equivalents (TE mmol/g).

### 2.5 CB1 and CB2 receptor agonist screening assay

Human embryonic kidney (HEK) 293T cells were seeded onto poly-F-lysine-coated 96-well plates at a density of 5 × 10^4^ cells/well. After 24 hours of incubation, cells were transfected with 1 ng of plasmids specifically designed for the study, using Lipofectamine 3000 (Invitrogen, Carlsbad, CA, USA) according to the manufacturer’s instructions. After 22 hours of transfection, the candidate compounds and Nano-Glo Live Cell Reagent were added to the cells in accordance with the manufacturer’s protocol (N2014, Promega, Madison, WI, USA). The luminescence was detected by ENSPIRE and ENVISI (PerkinElmer, Waltham, MA, USA) for 60 min, and the results were analyzed using GraphPad Prism software.

### 2.6 Statistical analysis

Principal component analysis (PCA) and orthogonal partial least-squares discriminant analysis (OPLS-DA) were conducted using SIMCA-P 14.0 software (Umerics, Umea, Sweden). Hierarchical cluster analysis (HCA) was performed on the online website (https://www.metaboanalyst.ca/). Heatmap was created using MeV software (Multiple ExperimentViewer, Version 4.9.0). One-way analysis of the variance (ANOVA) was performed using the SPSS software (version 22.0, International Business Machines Corporation, New York, NY, USA), followed by Tukey’s *post-hoc* test (*p* > 0.05). Pearson’s correlations (*p* < 0.05; two-tailed) were also computed using SPSS software.

## 3 Results

### 3.1 Metabolic profiling of 10 different parts of *P. ostii*

**Figure 1:**
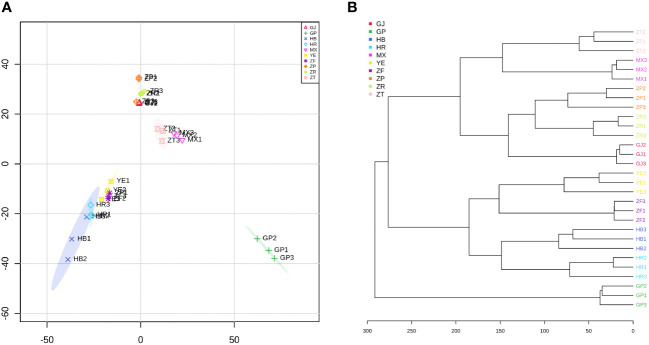
PCA and HCA of metabolic features detected in 10 different parts of *Paeonia ostii*. **(A)** PCA score plot. **(B)** HCA plot. GP, root bark; MX, root core; ZT, branches; YE, leaves; HB, petals; HR, stamens; ZF, ovary; ZP, seed coats; ZR, seed kernels; GJ, fruit shells; PCA, principal component analysis; HCA, hierarchical cluster analysis.

Untargeted metabolomics via UPLC-Q-TOF-MS technology provides a more comprehensive overview of the variations in all detected metabolites between different groups. **Figure S1** shows that there were significant differences in the base peak chromatograms of each plant part extract. To further elucidate the phytochemical differences between groups, the unsupervised PCA was initially conducted on 30 samples. As shown in **Figure 1A**, the first two principal components account for a total of 35.5% of the variance in the PCA plot. HCA (**Figure 1B**) showed analogous results to the PCA scoring plot and clearly distinguished each group. As an important medicinal part, the samples of root bark (GP) were analyzed separately and exhibited distinct differences in chemical composition compared with other parts. The study identified 55 compounds from *Paeonia suffruticosa* using LC-IT-TOF/MS technology and concluded that the chemical compositions of root core (MX) and root bark from *P. suffruticosa* were similar by comparing whether these compounds could be detected in these two plant parts (Pan et al., 2020). Instead, in the current study, the chemical composition of the root bark showed obvious differences compared to the root core. This finding suggested that untargeted metabolomics allowed a more comprehensive comparison of different samples by detecting a wide range of small molecules. Furthermore, PCA and HCA results indicated that branches (ZT) and root core had similar metabolic profiles. Three parts of the fruits (seed coats (ZP), seed kernels (ZR), and fruit shells (GJ)) and flowers (petals (HB), stamens (HR), and ovary (ZF)) and leaves (YE) overlapped and interacted together in the PCA. These samples also showed a certain tendency to separate from other samples. In the HCA plot, petals and stamens displayed similar chemical characteristics, and leaves and the ovary demonstrated a closer relationship based on the detected metabolites. In a word, the differences and similarities in the metabolic profiles of these 10 different parts of *P. ostii* suggest the presence of variations in their specific chemical composition.

### 3.2 Metabolite identification of 10 different parts of *P. ostii* based on UPLC-Q-TOF-MS

A comprehensive in-house database encompassing 464 compounds derived from the related literature on the phytochemistry of genus *Paeonia* was established and applied to identify compounds of *Sect. Moutan* in our previous research (Wang et al., 2019). Similarly, this database was used to identify the metabolic features of 10 different parts of *P. ostii* in the current study. The experimental fragments of features in MS1 and MS2 modes were compared with the theoretical fragments of compounds provided by Progenesis QI, and the identification results with high total and fragmentation scores were subsequently filtered and manually examined (Gu et al., 2019). A total of 160 metabolomic features, corresponding to 213 compounds, were tentatively identified. Among them, 26 compounds were identified through authentic standard comparison (level 1), while the remaining compounds were identified through an in-house database (level 2, i.e., putatively annotated compounds) (Sumner et al., 2007). The structures of the identified compounds were categorized into seven different types (33 flavonoids, 71 monoterpene glycosides, 26 phenols, 14 stilbenes, 25 tannins, 27 triterpenes, steroids, and 17 others). The comprehensive details of these compounds, including major ions, adducts, molecular formula, total score, fragmentation score, mass error, and isotope similarity, are shown in **Table S2**. Compounds with similar structures and MS^n^ spectra can potentially yield identical mass spectrometry fragments for multiple identification results for a single metabolic feature. Although the exact chemical structure may remain undetermined, it was possible to determine the chemical classes to which the metabolite belongs. There were several compounds that showed different additive-sum forms ([M–H]^−^, [2M–H]^−^, and [M+FA–H]^−^) in the detection. The different ions may have the same identification result. For instance, two features, 3.30_460.1576n and 3.42_505.1559m/z [M+FA–H]^−^, were both identified as apiopaeonoside, based on the experimental fragments they generated. Overall, this table of identification showed a clear differentiation between these metabolic features in terms of compound class.

### 3.3 Semi-quantitative analysis of compounds identified from 10 parts of *P. ostii*

**Figure 2:**
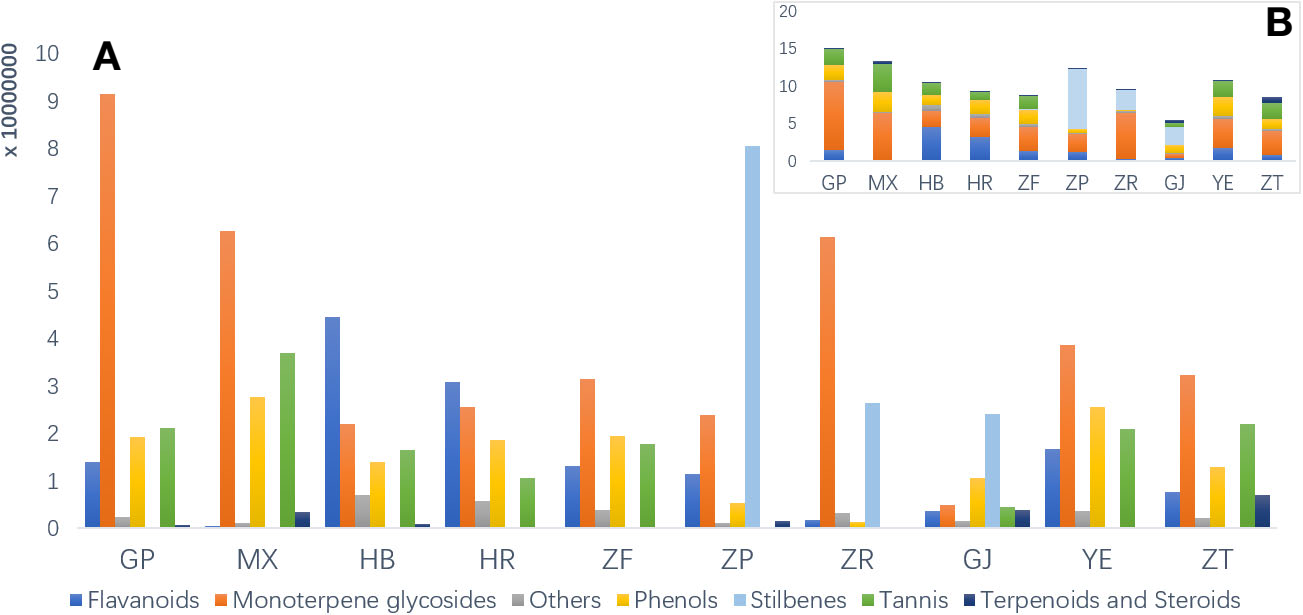
Raw peak abundance of different kinds of secondary metabolites in 10 different parts of *Paeonia ostii*. **(A)** Total raw abundance by different kinds of compounds in different plant parts. **(B)** An overview of total raw abundance. GP, root bark; MX, root core; ZT, branches; YE, leaves; HB, petals; HR, stamens; ZF, ovary; ZP, seed coats; ZR, seed kernels; GJ, fruit shells.

To investigate the disparities in metabolic profiles of 10 different parts of *P. ostii*, the identified metabolites were first analyzed semi-quantitatively based on their peak abundance. **Figure 2** illustrates the relative distribution of each identified phytochemical class in the analyzed samples. Monoterpene and derived glycosides possessing a “cage-like” pinane skeleton were the main chemical constituents, exhibiting the broadest distribution and highest abundance throughout all the 10 parts of *P. ostii*. For example, paeoniflorin, oxypaeoniflorin, albiflorin, and benzoylpaeoniflorin were reported to be the primary active ingredients responsible for the anti-inflammatory effects of *Paeonia* spp. (Li et al., 2021). As the most important medicinal part of *P. ostii*, the root bark exhibited the highest levels of monoterpene glycosides, followed by the root core, leaves, and branches, and the lower parts were the seed coats and fruit shells. The semi-quantitative results also demonstrated that root bark was the most diverse part of *P. ostii* in terms of compounds. Notably, the distribution of metabolites is closely related to the organs of plants. In the current study, the identified stilbenes in *P. ostii* were found to be oligomers of resveratrol. Analysis showed that they were most abundant in the seed coat, followed by the fruit shells and seed kernels. The content of stilbenes in these three parts was significantly higher compared to that of other parts of the plant, indicating a tissue-specific distribution of these compounds in the evaluated samples. These findings were in agreement with our summary in the previous review about the phytochemistry of genus *Paeonia* (Li et al., 2021). With respect to the flavonoids, most of them were identified as flavonol glycosides, as shown in **Table S2**. The concentrations of these compounds were higher in flowers (petals and HR) and YE than in other parts of the plant. The extracts of these flavonoid-rich parts have been reported to exhibit significant antioxidant activity (Bao et al., 2018). In addition to the fruit parts (seed coats and seed kernels), tannins are widely distributed in almost all other organs of *P. ostii*, with the highest content in the root core. Moreover, triterpenes and steroids are the least abundant of these compounds.

### 3.4 Identification of potential biomarkers based on multivariate statistical analysis for the 10 different parts of *P. ostii*

To compare the metabolites in underutilized plant parts with those in commonly used plant parts, OPLS-DA models and S-Plot were utilized to identify the marker features responsible for the chemical differences between these groups. The parameters of the OPLS-DA models are outlined in **Table S3**, and the high R2 and Q2 values obtained from each model indicated their excellent repeatability and predictive capabilities. The distinctive metabolic features that played a significant role in differentiating the 10 parts of *P. ostii* were selected as markers based on the variables represented by the ions located at both ends of the S-Plot (indicated by red circle marks in **Figure S2**). These markers were subsequently imported into Progenesis QI software for identification. In order to capture the differences between different plant parts as comprehensively as possible, a total of 45 potential pairwise combinations were compared among the 10 plant parts. Eight pairs were selected for analysis based on the proximity of the plant parts to each other. Subsequently, a total of 55 marker compounds were identified, as listed in **Table 1**. These marker compounds include 13 flavonoids, 15 monoterpene glycosides, four phenols, 11 stilbenes, nine tannins, and one triterpene.

**Table 1:** Tentative identification of potential biomarkers selected from 10 different parts of *Paeonia ostii* based on the S-Plot.

No.	Feature	Retention time (min)	m/z	Adducts/deprotonated ions	Fragment ions	Formula	Mass error (ppm)	Description	Class
1	1.13_332.0738n	1.13	331.0665	M–H, 2M–H	292,169	C_13_H_16_O_10_	−1.75	1-*O*-Galloyl-*β*-d-hexoside	Tannins
2	1.32_376.1362n	1.32	375.1289	M–H, M+FA–H, 2M–H	341,325,169	C_16_H_24_O_10_	−2.11	8-Debenzoylpaeoniflorin	Monoterpene glycosides
3	1.39_331.0664m/z	1.39	331.0664	M–H	271,169,125	C_13_H_16_O_10_	−1.89	1-*O*-Galloyl-*β*-d-hexoside	Tannins
4	1.42_169.0137m/z	1.42	196.0137	M–H	–	C_7_H_6_O_5_	0.00	Gallic acid*	Phenols
5	1.47_360.1413n	1.47	359.1340	M–H, M+FA–H, 2M–H	315,197	C_16_H_24_O_9_	−2.03	1-*O*-*β*-d-Glucopyranosylpaeonisuffrone	Monoterpene glycosides
6	2.56_496.1577n	2.56	495.1504	M–H, 2M–H	443,285	C_23_H_28_O_12_	−0.75	Paeonin C	Monoterpene glycosides
7	2.71_496.1573n	2.71	495.1500	M–H, M+FA–H, 2M–H	431,295,191	C_23_H_28_O_12_	−0.61	Oxypaeoniflorin*	Monoterpene glycosides
8	2.81_290.0785n	2.81	289.0713	M–H, 2M–H	245,179,137	C_15_H_14_O_6_	−1.74	Catechin*	Flavonoids
9	3.02_184.0369n	3.02	183.0296	M–H, 2M–H	168,139,124	C_8_H_8_O_5_	−1.57	Me gallate (Gallicin) *	Phenols
10	3.08_610.1525n	3.08	609.1452	M–H, 2M–H	541,495	C_27_H_30_O_16_	−1.51	Quercetin 3-galacto-7-methyl pentose	Flavonoids
11	3.26_642.2147n	3.26	687.2130	M–H, M+FA–H	641,459,362	C_29_H_38_O_16_	−2.06	6′-*O*-*β*-d-Glucopyranosylalbiflorin	Monoterpene glycosides
12	3.30_460.1576n	3.30	505.1558	M+FA–H	463,293	C_20_H_28_O_12_	−1.00	Apiopaeonoside*	Tannins
13	3.42_505.1559m/z	3.42	505.1559	M+FA–H	459,293	C_20_H_28_O_12_	−0.77	Apiopaeonoside*	Tannins
14	3.57_460.1575n	3.57	459.1503	M–H	293,233, 153	C_20_H_28_O_12_	0.00	Paeonolide*	Phenols
15	3.63_642.2150n	3.63	687.2132	M–H, M+FA–H	551,459,345	C_29_H_38_O_16_	−1.58	*β*-Gentiobiosylpaeoniflorin	Monoterpene glycosides
16	3.78_165.0551m/z	3.78	165.0551	M–H	112	C_9_H_10_O_3_	−3.74	2,5-Dihydroxy-4-methylacetophenone	Phenols
17	3.84_480.1627n	3.84	525.1609	M–H, M+FA–H, 2M–H	479,435,323	C_23_H_28_O_11_	0.19	Albiflorin*	Monoterpene glycosides
18	4.21_480.1624n	4.21	525.1606	M+FA–H, 2M–H, M–H	479,449	C_23_H_28_O_11_	−1.62	Paeoniflorin*	Monoterpene glycosides
19	4.32_640.1630n	4.32	639.1558	M–H, 2M–H	525,449	C_28_H_32_O_17_	−1.42	Sexangularetin-3-*O*-*β*-d-sophoroside	Flavonoids
20	4.57_670.1735n	4.57	669.1662	M–H, 2M–H	457,393,241	C_29_H_34_O_18_	−1.49	Limocitrin-3-*O*-yl-*β*-d-sophoroside	Flavonoids
21	5.35_448.0998n	5.35	447.0925	M–H, 2M–H	393,335,183	C_21_H_20_O_11_	−1.74	Astragalin/populnin/cynaroside	Flavonoids
22	5.69_480.1625n	5.69	525.1607	M+FA–H, 2M–H, M–H	449,335,183	C_23_H_28_O_11_	−1.29	Mudanpioside I	Monoterpene glycosides
23	6.02_1091.1216m/z	6.02	1,091.1220	M–H	1090,939,771,545	C_48_H_36_O_30_	−0.22	6-*O*-(*m*-Galloyl)galloyl-1,2,3,4-tetra-*O*-galloyl-*β*-d-hexose	Tannins
24	6.09_448.0997n	6.09	447.0924	M–H, 2M–H	–	C_21_H_20_O_11_	−1.91	Kaempferol-*O*-hexoside	Flavonoids
25	6.33_1091.1218m/z	6.33	1,091.1220	M–H	977,863,545,431	C_48_H_36_O_30_	−0.05	Isomer of 6-*O*-(*m*-galloyl)galloyl-1,2,3,4-tetra-*O*-galloyl-*β*-d-hexoside	Tannins
26	6.36_431.0977m/z	6.36	431.0977	M–H	271,195	C_21_H_20_O_10_	−1.44	Apigenin 7-*O*-hexoside	Flavonoids
27	6.84_287.0556m/z	6.84	287.0556	M–H	259	C_15_H_12_O_6_	−1.61	Dihydrokaempferol	Flavonoids
28	6.89_479.1551m/z	6.89	479.1551	M+FA–H	447,357,243	C_22_H_26_O_9_	−1.73	Lactiflorin	Monoterpene glycosides
29	7.69_680.2033n	7.69	679.1959	M–H, 2M–H	573,445,349	C_42_H_32_O_9_	−1.33	Suffruticosol A*	Stilbenes
30	7.84_600.1837n	7.84	599.1764	M–H, 2M–H	491,445,349	C_30_H_32_O_13_	−0.17	Mudanpioside C	Flavonoids
31	8.00_525.1607m/z	8.00	525.1607	M+FA–H	493,419,283	C_23_H_28_O_11_	−1.44	Floralalbiflorin I	Monoterpene glycosides
32	8.40_600.1835n	8.40	599.1762	M–H, M+FA–H, 2M–H	505,445,325	C_30_H_32_O_13_	−0.50	Benzoyl oxypaeoniflorin*	Flavonoids
33	8.53_680.2035n	8.53	725.2017	M+FA–H, 2M–H, M–H	679,585,453	C_42_H_32_O_9_	−1.62	Ampelopsin E	Stilbenes
34	8.72_286.0472n	8.72	285.0399	M–H, 2M–H	245,174,112	C_15_H_10_O_6_	−1.90	Luteolin	Flavonoids
35	8.72_630.1940n	8.72	629.1867	M–H, 2M–H, M+FA–H	505,447,285	C_31_H_34_O_14_	−1.33	3″-Methoxy-4″-hydroxy-6′-benzoyl-paeoniflorin	Monoterpene glycosides
36	8.95_680.2035n	8.95	679.1962	M–H	601,469	C_42_H_32_O_9_	−0.88	Suffruticosol B*	Stilbenes
37	9.43_680.2030n	9.43	679.1957	M–H, M+FA–H, 2M–H	677,529,453	C_42_H_32_O_9_	−1.62	Suffruticosol C*	Stilbenes
38	10.32_454.1408n	10.32	453.1336	M–H, M+FA–H, 2M–H	311,271,183	C_28_H_22_O_6_	−0.44	*cis*-Viniferin*	Stilbenes
39	10.38_584.1885n	10.38	584.1885	M+FA–H, 2M–H, M–H	553,453,399,269	C_30_H_32_O_12_	−1.53	Benzoylwurdin	Monoterpene glycosides
40	10.39_269.0449m/z	10.39	269.0449	M–H	230,196,149	C_15_H_10_O_5_	−2.26	Apigenin	Flavonoids
41	10.60_679.1960m/z	10.60	679.1960	M–H	529,361	C_42_H_32_O_9_	−1.18	*cis*-Suffruticosol D*	Stilbenes
42	10.69_614.1994n	10.69	659.1973	M–H, M+FA–H	629,553,341	C_31_H_34_O_13_	−0.89	4-*O*-Methylbenzoyloxypaeoniflorin	Monoterpene glycosides
43	10.74_285.0398m/z	10.74	285.0398	M–H	242,174,146	C_15_H_10_O_6_	−0.35	Kaempferol*	Flavonoids
44	10.74_679.1958m/z	10.74	679.1958	M–H, M+FA–H, 2M–H	629,599,391	C_42_H_32_O_9_	−1.47	*cis*-Gnetin H*	Stilbenes
45	11.06_677.1806m/z	11.06	677.1806	M+FA–H	583,449,323,257	C_30_H_32_O_15_	13.06	4-*O*-Galloylalbiflorin	Monoterpene glycosides
46	11.19_680.2035n	11.19	679.1963	M–H, M+FA–H	607,513,385	C_42_H_32_O_9_	−0.74	*trans*-Suffruticosol D*	Stilbenes
47	11.23_454.1412n	11.23	453.1337	M–H, M+FA–H, 2M–H	285	C_28_H_22_O_6_	−0.22	*trans*-Viniferin*	Stilbenes
48	11.60_165.0551m/z	11.60	165.0551	M–H	150,112	C_9_H_10_O_3_	−0.61	Paeonol*	Phenols
49	11.83_679.1963m/z	11.83	679.1963	M–H	511,329	C_42_H_32_O_9_	−0.74	*trans*-Gnetin H*	Stilbenes
50	11.88_725.2017m/z	11.88	725.2017	M+FA–H	679,552,480	C_42_H_32_O_9_	−1.65	Rockiiol C	Stilbenes
51	12.23_287.2221m/z	12.23	287.2221	M+FA–H	183,112	C_15_H_30_O_2_	−2.62	13-Methyltetradecanoic acid	Others
52	12.68_358.2137n	12.68	357.2064	M–H, 2M–H	325,292,183	C_22_H_30_O_4_	−2.02	Palbinone	Terpenoids and steroids
53	2.39_577.1345m/z	2.39	577.1345	M–H	483,425,389,343	C_30_H_26_O_12_	−1.11	Procyanidin B-7/procyanidin B-3	Tannins
54	5.11_442.0891n	5.11	442.0891	M–H, 2M–H	441,335,271	C_22_H_18_O_10_	−1.97	Catechin-7-*O*-gallate	Tannins

* Identifications were confirmed by comparing tR and MS spectra to standard compounds.

**Figure 3:**
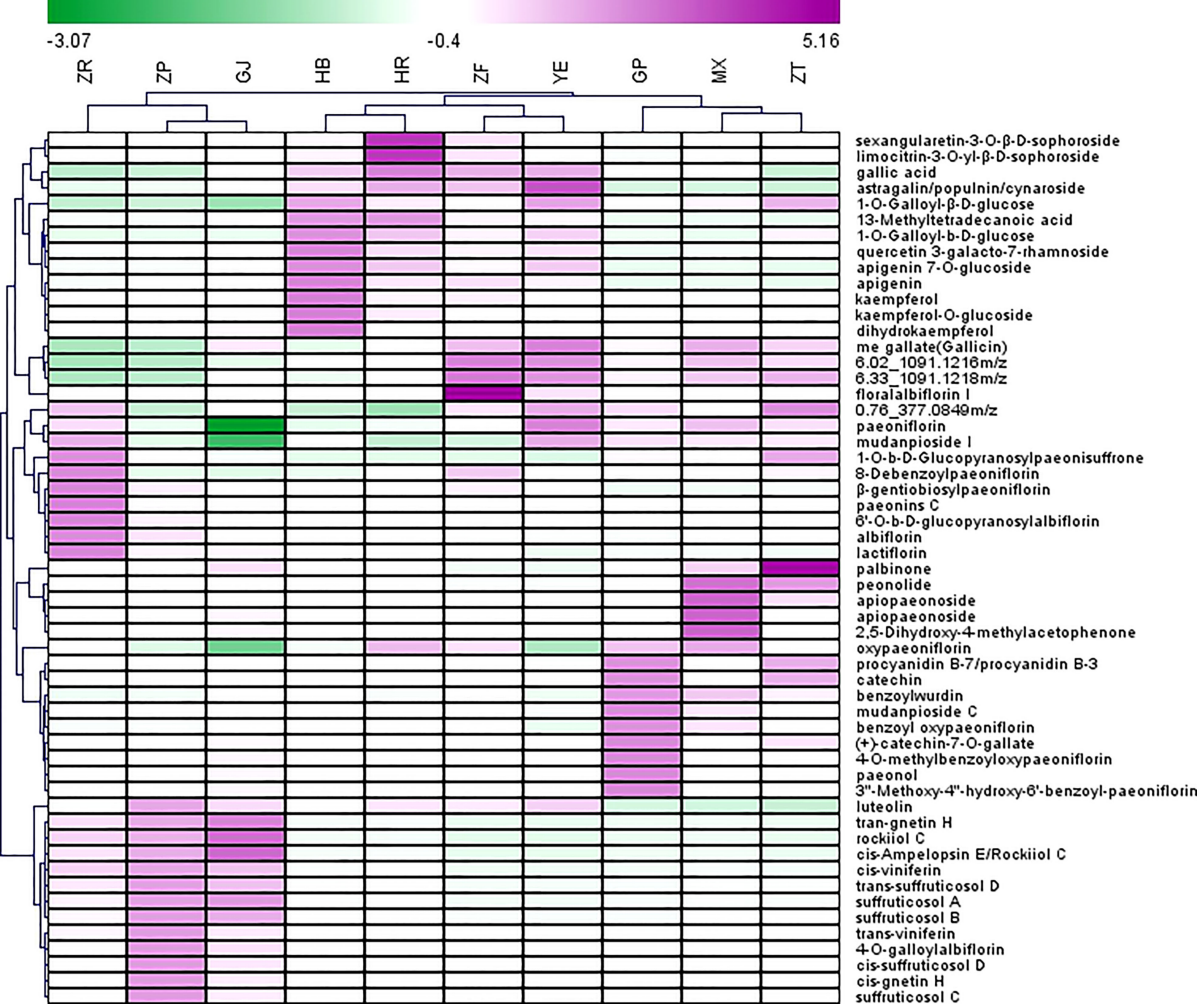
Heatmap of top 55 potential biomarkers selected from 10 different parts of *Paeonia ostii* based on the S-Plot. GP, root bark; MX, root core; ZT, branches; YE, leaves; HB, petals; HR, stamens; ZF, ovary; ZP, seed coats; ZR, seed kernels; GJ, fruit shells.

Heatmap was used to visualize the distribution of the contents of identified marker compounds in 10 plant parts of *P. ostii*. As depicted in **Figure 3**, the characteristic compounds of root bark are mainly monoterpene glycosides, such as oxypaeoniflorin, benzoyl oxypaeoniflorin, benzoylpaeoniflorin, and paeoniflorin. Moreover, as an important bioactive compound, it is reported that paeonol was more abundant in roots than other plant organs (including leaves, petals, petiole, and stem) of *P. ostii* (Zhang et al., 2019). The same results were obtained in the current study; i.e., paeonol was much more abundant in GP (root phloem) and MX (root xylem) than in the other eight plant parts of *P. ostii*. These results indicate that the root core also has the potential as a valuable medicinal resource (Wang et al., 2017b; Wang et al., 2018). We can find that the root core and branches demonstrated similar metabolic profiles as determined by HCA and PCA. In comparison to the root bark, the root core contained higher levels of methyl gallate, as well as high levels of paeonolactone and apiopaeonoside. However, branches had high contents of catechin derivatives (catechin-7-*O*-gallate, procyanidins, etc.) and palbinone, which was a triterpenoid compound that has been shown to have good anti-inflammatory activity and had the effect of improving liver fibrosis and retinopathy (Li et al., 2023). Furthermore, previous research also reported that *P. ostii* seed kernels contained high levels of (+)-catechin and (−)-catechin (Zhang X, et al., 2017). However, these compounds were not detected in the current study, which could potentially be attributed to variations in the extraction methods used for the samples.

The main characteristic compounds found in the fruit were stilbenes, being the most representative ingredients in this organ; these resveratrol oligomers had the highest content and were most diverse in seed coat extracts. These resveratrol oligomers have demonstrated various biological activities, such as anti-microbial, anti-tumor, and antioxidant activities (Shen et al., 2017). Our previous research demonstrated the neuroprotective properties of resveratrol oligomers extracted from *P. suffruticosa* seed coats (Liu et al., 2020). Another study has also reported that resveratrol oligomer compounds extracted from *Paeonia veitchii* Lynch seed coats exhibit antibacterial and moderate anticancer activities (Zhang et al., 2018). In recent years, attention has been focused on seed coats to isolate new stilbene natural products and evaluate their biological properties, and the fruit shells of *P. ostii* with huge resources remained neglected. As is shown in **Figure 2**, fruit shells also contained a higher content of stilbenes than other plant parts. As by-products of peony seed oil production, both seed coats and fruit shells possess significant potential for applications in the biomedical and pharmaceutical industries.

Due to the limitation of the extraction and analysis methods, UFA was not detected in the seed kernel extracts in the current study. However, it is worth noting that the chemical composition of seed kernel extracts in the current study is similar to the seed kernel residue (also called peony seed meal or seed cakes) after the edible oil extraction. As is shown in **Figure 2**, compared with root bark and fruit shells, seed kernel extracts (seed cakes) contain higher contents of monoterpene glycosides, including paeonin C, albiflorin, and lactiflorin. In addition to these compounds, previous studies have reported the presence of plant proteins in oil peony seed cakes (Liu et al., 2018; Ruixue et al., 2019). With the increasing area of oil peony cultivation, it is expected that in 5–10 years, more than 570,000 tons of residues of peony seed oil will be produced annually (Ruixue et al., 2019). Taking into account their rich composition, oil peony seed cakes present various potential applications. Considering the rich constituents, oil peony seed cakes can be utilized for isolating bioactive monoterpene glycosides or as a basis for developing health supplements for human consumption, such as protein drinks and powders. Moreover, oil peony seed cakes hold promise in the field of animal husbandry. By using them, organic feeds with low antibiotic contents can be produced, contributing to healthier and more sustainable livestock feeding practices. Furthermore, these seed cakes can be employed as bio-organic fertilizers, benefiting the agricultural industry and promoting environmentally friendly cultivation methods.

With regard to extracts from flowers (petals, stamens, and ovary) and leaves, flavonoids and derived glycosides and tannins are the main characteristic metabolites. Consistent with previous findings, the flower extracts were rich in flavonoids (Zhang H, et al., 2017). Wang et al. investigated the contents of flavonoids in the petals of 39 tree peony cultivars; kaempferol, apigenin, and their derivatives were the main flavonoid compounds in *P. ostii* petals (Wang et al., 2004). In the present study, it was observed that the metabolic profiles of petals and the ovary were quite similar. However, the analysis revealed that flavonol glycosides with a kaempferol skeleton, such as kaempferol, kaempferol-*O*-glucoside, and dihydrokaempferol, as well as flavones like apigenin and apigenin 7-*O*-glucoside, were more abundant in the petals compared to the stamens. Anthocyanins also are important flavonoid ingredients in peony flowers. Only one anthocyanin, peonidin, was identified in this study. The content of peonidin was low, which is probably due to the petals collected in this study being white. It is reported that the petal color and anthocyanin content of *P. ostii* are closely correlated, and *P. ostii* with white petals contains almost no anthocyanins (Gao et al., 2016). Compared to the petal extracts, stamen extracts contained higher levels of sophoroside flavonols (sexangularetin-3-*O*-*β*-d-sophoroside and limocitrin-3-*O*-yl-*β*-d-sophoroside) and tannins (such as methyl gallate and 1-*O*-galloyl-*β*-d-glucose). Notably, the ovary extracts also had high levels of tannin compounds, including methyl gallate and 6-*O*-(*m*-galloyl)galloyl-1,2,3,4-tetra-*O*-galloyl-β-d-glucose, and they also contain surprisingly high concentrations of monoterpene glycosides: floralalbiflorin I. In this study, the leaves also contain higher contents of monoterpene glycosides (albiflorin, paeoniflorin, floralalbiflorin, and mudanpioside I) compared to the flowers.

### 3.5 Quantitative analysis of important biomarkers in 10 parts of *P. ostii*

In order to quantitatively analyze the significant potential bioactive components in various parts of *P. ostii*, 28 compounds were determined by UPLC-DAD technology based on the method previously established for the experiment, and the findings are presented in **Table 2**. The highest content of gallic acid was detected in the leaves (3.12 ± 0.14 mg/g), followed by the stamens (1.82 ± 0.05 mg/g) and the petals (1.80 ± 0.07 mg/g). Gallic acid was also found to be present mainly in leaf sites in previous studies (Pan et al., 2020). Methyl gallate was the most abundant in the ovary (17.26 ± 0.70 mg/g) and was also present in the leaves (8.63 ± 0.19 mg/g), root core (6.19 ± 0.41 mg/g), and stamens (5.77 ± 0.07 mg/g). However, the level of catechin was the highest in the root bark (2.20 ± 0.05 mg/g). Minor quantities of catechin were detected in the stamens, ovary, leaves, and branches (0.29~0.52 mg/g), while catechin was not detected within several other parts. Oxypaeoniflorin was not detected in the seed coats and fruit shells, but it exhibited varying levels (0.64~3.68 mg/g) in all other parts, with the highest content in the root bark. Paeonolide and apiopaeonoside were mainly present in the root bark (17.91 ± 3.41 mg/g and 67.52 ± 2.17 mg/g, respectively) and root core (12.49 ± 0.57 mg/g and 3.50 ± 0.24 mg/g, respectively). Albiflorin had the highest content in leaves (5.86 ± 0.10 mg/g). It was also detected in petals (2.00 ± 0.03 mg/g), stamens (2.02 ± 0.02 mg/g), and seed kernels (1.59 ± 0.04 mg/g), but it was not found in the root bark. Paeoniflorin, however, is considered the main active component in *Cortex Moutan*, which is also one of the critical index components for quality control of *Cortex Moutan*. However, interestingly, in the experiment, the leaves exhibited the greatest paeoniflorin content (38.68 ± 0.76 mg/g), followed by the root bark (25.44 ± 0.47 mg/g) and ovary (25.12 ± 0.45 mg/g), and all other parts also contained different degrees, except for the seed coats. Benzoic acid was the highest in the ovary (0.77 ± 0.18 mg/g). In the root bark, galloylpaeoniflorin has the most content (4.43 ± 0.42 mg/g), and it is also higher in the root core (1.87 ± 0.03 mg/g) and stamens (2.82 ± 0.20 mg/g). PGG is an active hydrolyzable tannin, with high content in most parts of *P. ostii*. The ovary exhibited the highest content of PGG (38.14 ± 1.27 mg/g), followed by the root bark (25.18 ± 0.51 mg/g), stamens (18.97 ± 0.72 mg/g), and leaves (16.32 ± 0.87 mg/g). Quercitrin and quercetin are flavonoids and chromogenic substances. Quercitrin was predominantly present in the petals (6.01 ± 0.04 mg/g), quercetin was found in smaller amounts (0.66 ± 0.12 mg/g), and kaempferol was either not detected or fell below the detection limit in almost all parts of the plant. Mudanpioside C was mainly detected in the root bark (0.80 ± 0.51 mg/g) and root core (0.81 ± 0.46 mg/g), with smaller amounts observed in the stamens (0.39 ± 0.10 mg/g). Consistent with the previous studies (Pan et al., 2020), benzoyl oxypaeoniflorin and benzoylpaeoniflorin, as analogs of paeoniflorin, were both mainly distributed in the root bark part, with contents of 1.09 ± 0.09 mg/g and 5.45 ± 0.07 mg/g, respectively. Paeonol is the main active ingredient of *Cortex Moutan*, which is another indicator component of its quality control. It mainly existed in the root bark (21.75 ± 1.55 mg/g), with smaller amounts present in the root core (3.67 ± 0.16 mg/g) and petals (0.08 ± 0.01 mg/g). Among 10 stilbenes, *trans*-resveratrol, *cis*-*ϵ*-viniferin, and *cis*-suffruticosol D were not detected in any part of the plant in this experiment. The remaining seven components were only distributed in the seed coats. Except for the relatively low content of *cis*-gnetin H (0.63 ± 0.10 mg/g), the other six components were fairly high in the seed coats. *trans*-Gnetin H (81.34 ± 1.30 mg/g) and suffruticosol B (70.11 ± 1.12 mg/g) were the two highest components, which aligns with previous findings reported in our laboratory (He et al., 2016).

**Table 2:** Quantitative analysis of constituents in *Paeonia ostii* (per dry weight, mg/g).

Compounds	Root bark (GP)	Root core (MX)	Petals (HB)	Stamens (HR)	Ovary (ZF)	Seed coats (ZP)	Seed kernels (ZR)	Fruit shells (GJ)	Leaves (YE)	Branches (ZT)
Gallic acid	1.26 ± 0.07	0.58 ± 0.03	1.80 ± 0.07	1.82 ± 0.05	0.81 ± 0.15	ND	0.54 ± 0.00	0.62 ± 0.09	3.12 ± 0.14	0.95 ± 0.05
Methyl gallate	3.19 ± 0.23	6.19 ± 0.41	2.00 ± 0.43	5.77 ± 0.07	17.26 ± 0.70	ND	0.38 ± 0	2.46 ± 0.11	8.63 ± 0.19	3.38 ± 0.19
Oxypaeoniflorin	5.95 ± 0.02	3.13 ± 0.02	2.30 ± 0.04	3.68 ± 0.04	3.28 ± 0.06	ND	0.90 ± 0.01	ND	1.19 ± 0.03	0.64 ± 0.01
Catechin	2.20 ± 0.05	ND	ND	0.29 ± 0.01	0.49 ± 0.12	ND	ND	ND	0.52 ± 0.07	0.40 ± 0.02
Paeonolide	17.91 ± 3.41	12.49 ± 0.57	ND	1.26 ± 0.09	ND	ND	ND	ND	ND	ND
Apiopaeonoside	67.52 ± 2.17	3.50 ± 0.24	ND	ND	ND	ND	ND	ND	ND	ND
Albiflorin	ND	ND	2.00 ± 0.03	2.02 ± 0.02	ND	ND	1.59 ± 0.04	ND	5.86 ± 0.10	ND
Paeoniflorin	25.44 ± 0.47	17.60 ± 0.06	8.03 ± 0.08	5.92 ± 0.05	25.12 ± 0.45	ND	15.72 ± 0.41	0.42 ± 0.01	38.68 ± 0.76	13.27 ± 0.45
Benzoic acid	Tr	0.25 ± 0.02	0.26 ± 0.03	0.32 ± 0.03	0.77 ± 0.18	ND	0.47 ± 0.02	ND	Tr	ND
Galloylpaeoniflorin	4.43 ± 0.42	1.87 ± 0.03	0.78 ± 0.26	2.82 ± 0.20	ND	ND	ND	ND	ND	1.03 ± 0.08
PGG	25.18 ± 0.51	15.50 ± 2.00	9.22 ± 0.57	18.97 ± 0.72	38.14 ± 1.27	ND	ND	3.82 ± 0.46	16.32 ± 0.87	5.24 ± 0.42
Quercitrin	Tr	0.62 ± 0.05	6.01 ± 0.04	1.34 ± 0.03	0.63 ± 0.16	ND	ND	0.73 ± 0.13	1.21 ± 0.08	0.66 ± 0.03
Mudanpioside C	0.80 ± 0.51	0.81 ± 0.46	Tr	0.39 ± 0.10	ND	ND	ND	ND	ND	Tr
Benzoyl oxypaeoniflorin	1.09 ± 0.09	0.27 ± 0.01	0.36 ± 0.06	ND	ND	ND	ND	Tr	ND	ND
Quercetin	ND	ND	0.66 ± 0.12	ND	ND	ND	ND	ND	ND	ND
Benzoylpaeoniflorin	5.45 ± 0.07	1.03 ± 0.04	ND	ND	ND	0.42 ± 0.18	ND	0.53 ± 0.03	ND	Tr
Kaempferol	Tr	ND	Tr	ND	ND	Tr	ND	Tr	ND	ND
Paeonol	21.75 ± 1.55	3.67 ± 0.16	0.08 ± 0.01	ND	ND	ND	ND	ND	ND	ND
Suffruticosol A	ND	ND	ND	ND	ND	24.92 ± 0.54	ND	ND	ND	ND
Suffruticosol B	ND	ND	ND	ND	ND	70.11 ± 1.12	ND	ND	ND	ND
Suffruticosol C	ND	ND	ND	ND	ND	8.47 ± 0.56	ND	ND	ND	ND
*trans*-Resveratrol	ND	ND	ND	ND	ND	ND	ND	ND	ND	ND
*cis*-*ϵ*-Viniferin	ND	ND	ND	ND	ND	ND	ND	ND	ND	ND
*trans*-*ϵ*-Viniferin	ND	ND	ND	ND	ND	12.74 ± 1.08	ND	ND	ND	ND
*cis*-Suffruticosol D	ND	ND	ND	ND	ND	ND	ND	ND	ND	ND
*cis*-Gnetin H	ND	ND	ND	ND	ND	0.63 ± 0.10	ND	ND	ND	ND
*trans*-Suffruticosol D	ND	ND	ND	ND	ND	20.68 ± 0.67	ND	ND	ND	ND
*trans*-Gnetin H	ND	ND	ND	ND	ND	81.34 ± 1.30	ND	ND	ND	ND

a Content values are expressed as the mean ± SD (n = 3).

b Tr: below quantitative limit.

c ND: not detectable.

### 3.6 Biological activity evaluation

#### 3.6.1 Extraction rate evaluation

**Figure 4:**
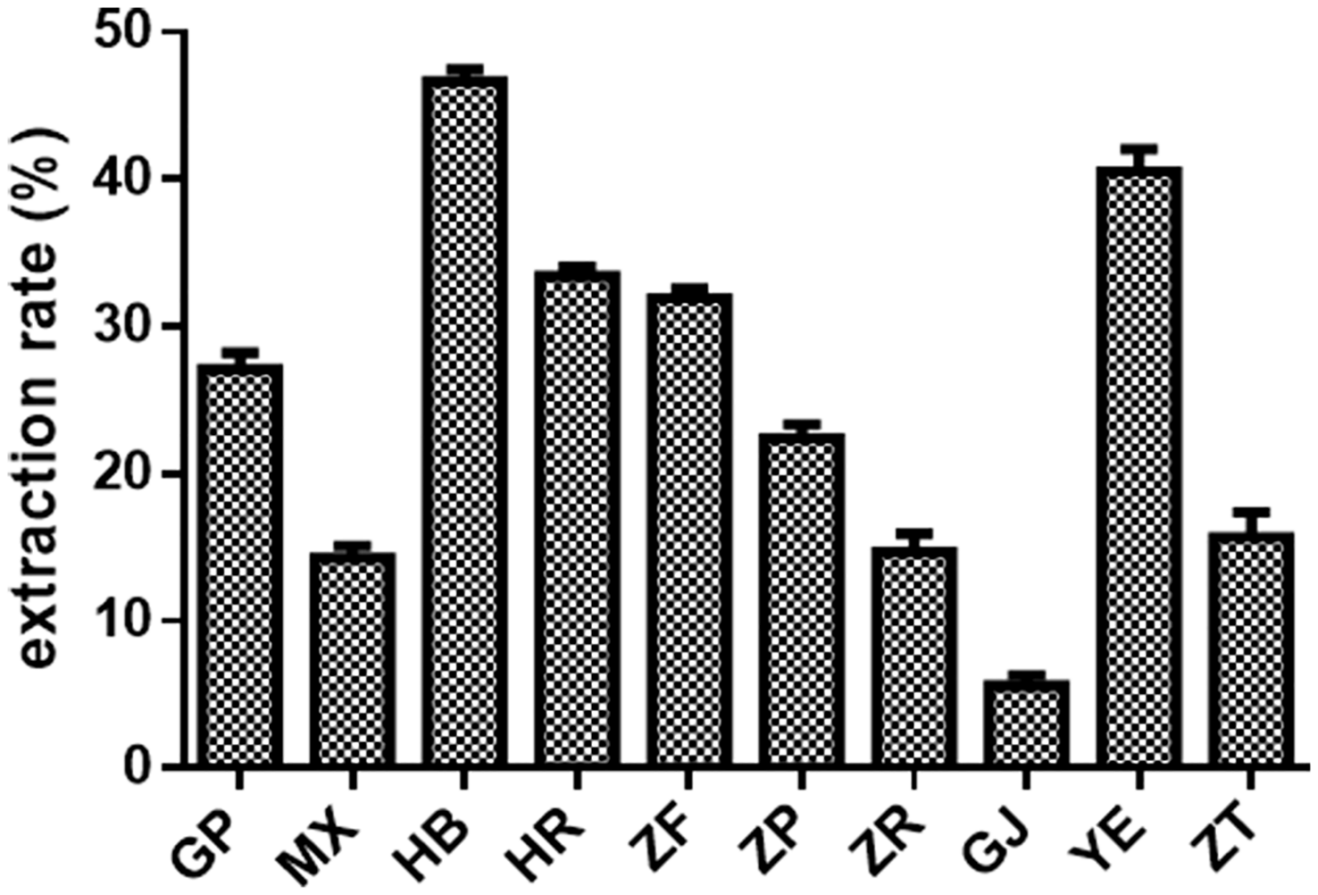
The extraction rates of 10 different parts of *Paeonia ostii*. GP, root bark; MX, root core; ZT, branches; YE, leaves; HB, petals; HR, stamens; ZF, ovary; ZP, seed coats; ZR, seed kernels; GJ, fruit shells.

The extraction rate is a basic index reflecting the effective utilization of samples. **Figure 4** illustrates the extraction rates of the powdered samples from 10 different parts of *P. ostii* (70% methanol–water was used as a solvent). The extraction rate for each plant part of the samples was as follows: petals > leaves > stamens > ovary > root bark > seed coats > branches > root core > seed kernels > fruit shells. Among the underground parts, the extraction rate of the root bark was significantly higher than that of the root core, which corresponds to the medicinal use of root bark in many traditional medicine systems such as *Cortex Moutan*. Regarding the aerial parts, both petals and leaves exhibited extraction rates that were notably higher compared to other parts, reaching over 30%. Seed coats were particularly rich in stilbenes, and their extraction rate was higher than that of seed kernels and fruit shells. *P. ostii* is a perennial woody plant, and its root bark is typically harvested as a medicinal part after 3–5 years of growth. During the growth process of the plant, the flowers, leaves, branches, fruit shells, and seed coats are renewable resources with high extraction rates, which should be fully utilized with appropriate theoretical guidance.

#### 3.6.2 Antioxidant activity evaluation

**Figure 5:**
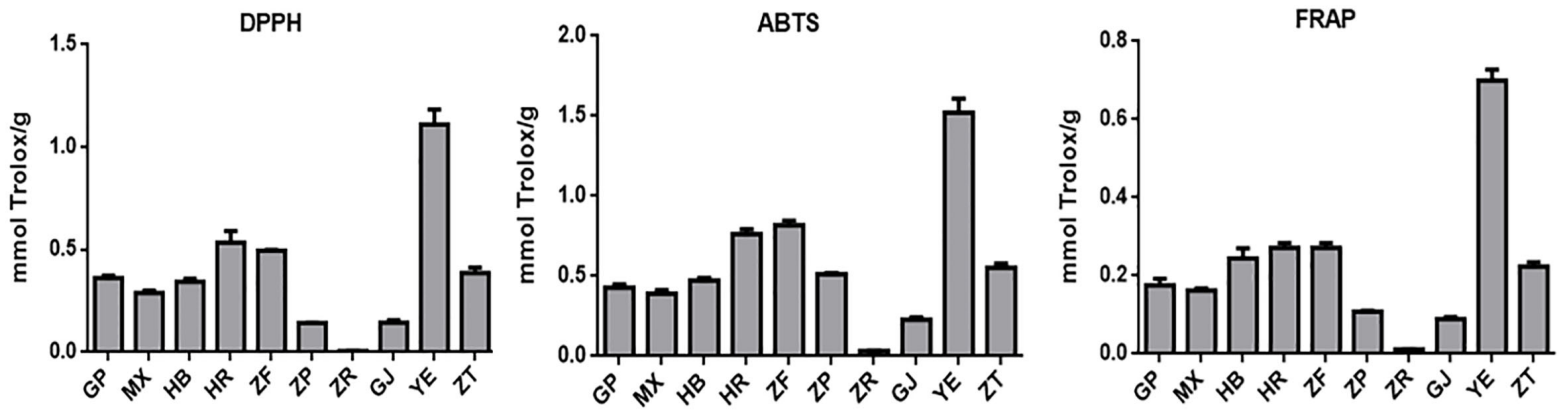
Antioxidant activity evaluation of 10 different plant part extracts obtained from *Paeonia ostii*. GP, root bark; MX, root core; ZT, branches; YE, leaves; HB, petals; HR, stamens; ZF, ovary; ZP, seed coats; ZR, seed kernels; GJ, fruit shells.

Oxidative stress is a condition characterized by an imbalance between oxidation and antioxidant action within the body. It is considered a significant factor in aging, neurodegenerative diseases, asthma, and cardiovascular dysfunction (Sindhi et al., 2013). Natural antioxidants developed from plants have received much attention due to their safety and effectiveness. To characterize the antioxidant properties of 70% methanol–water extracts obtained from *P. ostii* different parts, we tested the antioxidant capacity of evaluated samples using three different methods (DPPH, ABTS, and FRAP), and Trolox was used as the positive drug. The results obtained from the three different antioxidant systems (**Figure 5** and **Table 3**) indicate that the plant extracts from *P. ostii* generally exhibited modest antioxidant capacity, which showed higher antioxidant activity (0.09–1.52 mmol TE/g), except for the extracts from seed kernels. Based on the experimental results, antioxidant activity varied widely among different botanical organs of *P. ostii*. Leaves exhibited the highest antioxidant properties, and the flowers (petals, stamens, and ovary) also showed stronger antioxidant capacity than other parts. Previous studies have reported that the antioxidant activity of flower and leaf extracts obtained from *Paeonia* plants is closely associated with their high content of flavonoids and tannins (Bao et al., 2018; Dienaitė et al., 2019). Previous research has indicated that monoterpene glycosides in *Paeonia lactiflora* demonstrate significant antioxidant activity (Parker et al., 2016). Consistently, in this study, by chemical composition content determination, we observed that the parts exhibiting higher levels of monoterpene glycosides generally also displayed strong antioxidant activity. Moreover, the branches and root core, which had similar chemical components and were usually discarded after obtaining the medicinal parts (root bark), also had the potential of being resources of antioxidants. In the case of the fruit of *P. ostii*, the antioxidant activity of the seed coats and fruit shells was found to be higher compared to that of the seed kernel extracts (peony seed cakes). This difference could be attributed to the abundant presence of resveratrol oligomers in seed coats and fruit shells. However, it is worth noting that the antioxidant activity of the seed kernel extracts (peony seed cakes) was relatively weak in this study. This could be due to the limitations of the extraction method used, which failed to extract the UFAs known for their antioxidant effects from the seed kernels. The accumulation of phytochemicals was nfluenced by seasonal variations. Although the samples of different parts in this experiment were collected in different growing periods, their good antioxidant effects indicated the potential of these parts to be exploited as antioxidants.

**Table 3:** Antioxidant activity evaluation of 10 different plant part extracts obtained from *Paeonia ostii*.

Sample	DPPH (mmol Trolox/g)	ABTS (mmol Trolox/g)	FRAP (mmol Trolox/g)
Root bark	0.36 ± 0.01 cd	0.43 ± 0.01 cd	0.18 ± 0.01 c
Root core	0.29 ± 0.01 c	0.39 ± 0.02 c	0.16 ± 0.00 c
Petals	0.34 ± 0.01 cd	0.47 ± 0.01 cde	0.24 ± 0.02 de
Stamens	0.53 ± 0.05 e	0.76 ± 0.02 f	0.27 ± 0.01 e
Ovary	0.50 ± 0.00 e	0.82 ± 0.02 f	0.27 ± 0.01 e
Seed coats	0.14 ± 0.00 b	0.51 ± 0.01 de	0.11 ± 0.00 b
Seed kernels	0.01 ± 0.00 a	0.03 ± 0.00 a	0.01 ± 0.00 a
Fruit shells	0.14 ± 0.01 b	0.23 ± 0.01 b	0.09 ± 0.00 b
Leaves	1.11 ± 0.06 f	1.52 ± 0.07 g	0.70 ± 0.02 f
Branches	0.39 ± 0.02 d	0.55 ± 0.02 e	0.22 ± 0.01 d

Different letters (a–g) within the same column indicate significant differences based on Tukey’s multiple comparisons (p < 0.05).

FRAP, ferric ion reducing antioxidant power.

In this study, a total of 26 compounds were identified in *P. ostii*, including seven monoterpene glycosides, four flavonoids, three phenols, 10 stilbenes, and two tannins (level 1 identification). **Table 4** contains Pearson’s correlation coefficients (r) of the relative contents of these compounds (peak abundance of these compounds in 10 different parts) with antioxidant activity of extracts from 10 parts of *P. ostii*. Among these compounds, the highest correlation was observed with the content of compound T2 (PGG) in all three antioxidant evaluation systems, with correlation coefficients of r = 0.840, r = 0.814, and r = 0.820 in DPPH, ABTS, and FRAP, respectively. Compound S7 (*trans*-resveratrol) showed a positive correlation with antioxidant capacity in the DPPH and FRAP testing systems (r = 0.653 and r = 0.716, respectively). Additionally, the relative content of compound P3 (gallic acid) showed a positive correlation with the antioxidant activity of the tested samples (r = 0.624) in the DPPH assay.

**Table 4:** Pearson’s correlation values of 26 different types of compounds and the content and antioxidant activity of 10 different parts of *Paeonia ostii*.

No.	Feature	Identification	DPPH	ABTS	FRAP
Monoterpene glycosides					
M1	11.88_583.1814m/z	Benzoylpaeoniflorin	−0.051	−0.165	−0.113
M2	2.71_496.1573n	Oxypaeoniflorin	−0.064	−0.158	−0.178
M3	3.84_480.1627n	Albiflorin	−0.539	−0.447	−0.479
M4	4.21_480.1624n	Paeoniflorin	−0.359	0.314	0.358
M5	5.58_632.1733n	Galloylpaeoniflorin	0.068	0.530	0.565
M6	7.84_600.1837n	Mudanpioside C	−0.051	−0.165	−0.117
M7	8.40_600.1835n	Benzoyl oxypaeoniflorin	−0.048	−0.168	−0.119
Flavonoids					
F1	10.74_285.0398m/z	Kaempferol	0.005	−0.036	0.048
F2	6.29_448.1000n	Quercitrin	0.113	0.251	0.108
F3	2.81_290.0785n	Catechin	−0.045	−0.087	0.013
F4	8.79_302.0420n	Quercetin	0.200	0.132	0.160
Phenols					
P1	11.60_165.0551m/z	Paeonol	−0.032	−0.137	−0.092
P2	6.36_121.0291m/z	Benzoic acid	−0.128	−0.104	−0.177
P3	1.42_169.0137m/z	Gallic acid	**0.624**	0.571	0.584
Stilbenes					
S1	10.32_454.1408n	*cis*-Viniferin	−0.482	−0.281	−0.405
S2	10.60_679.1960m/z	*cis*-Suffruticosol D	−0.327	−0.104	−0.259
S3	10.74_679.1958m/z	*cis*-Gnetin H	−0.302	−0.078	−0.236
S4	11.19_680.2035n	*trans*-Suffruticosol D	−0.440	−0.229	−0.364
S5	11.23_454.1412n	*trans*-Viniferin	−0.358	−0.139	−0.288
S6	11.83_679.1963m/z	*trans*-Gnetin H	−0.485	−0.283	−0.407
S7	6.96_273.0762m/z	*trans*-Resveratrol	**0.653**	0.584	**0.716**
S8	7.69_680.2033n	Suffruticosol A	−0.502	−0.303	−0.422
S9	8.95_680.2035n	Suffruticosol B	−0.408	−0.193	−0.334
S10	9.43_680.2030n	Suffruticosol C	−0.308	−0.085	−0.242
Tannins					
T1	3.57_460.1575n	Paeonolide	−0.091	−0.154	−0.116
T2	5.86_939.1105m/z	PGG	**0.840**	**0.814**	**0.820**

FRAP, ferric ion reducing antioxidant power.

The bold values are the Pearson's correlation coefficients (r) of compounds T2, S7, and P3.

**Figure 6:**
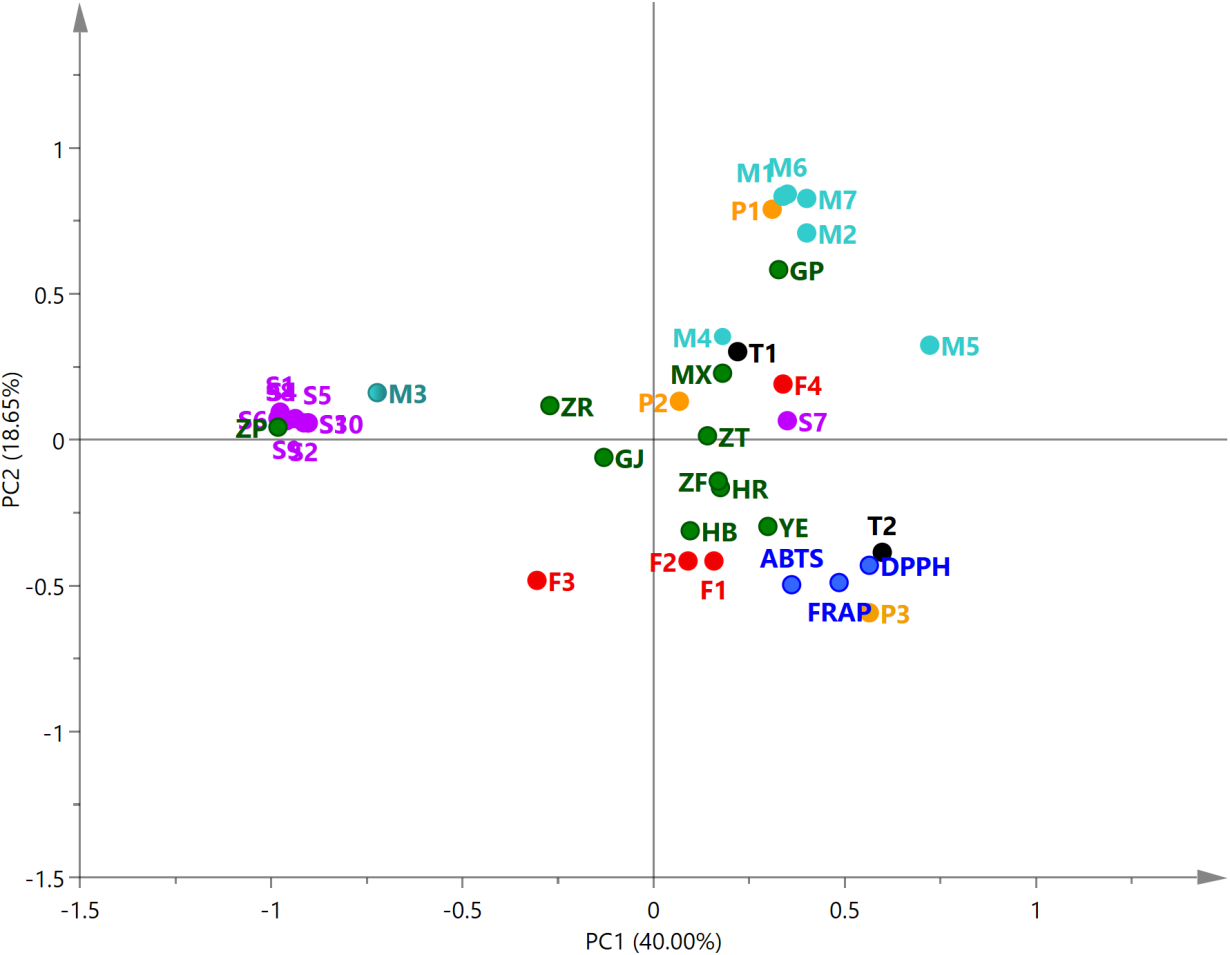
Principal component analysis biplot of 26 compounds and antioxidant activity for extracts from 10 parts of *Paeonia ostii*. M1–M7, monoterpene glycosides; F1–F4, flavonoids; P1–P4, phenols; S1–S10, stilbenes; T1–T2, tannins. Compound numbers correspond to those in **Table 4**.

PCA was performed on the antioxidant capacity of different parts of *P. ostii* and the relative content of 26 compounds. The relationships among the variables were shown in the biplot (**Figure 6**). The first two principal components (PC1, 40.00%; PC2, 18.65%) explained 58.65% of the total variables. The closer the distance of these variables in the biplot, the stronger the correlation between them (Karolina et al., 2021). From the analysis, it can be observed that the root bark extracts demonstrated a positive correlation with the relative content of paeonol and six monoterpene glycosides. However, the seed coat extracts were positively correlated with the 10 identified stilbenes. Leaves, petals, stamens, and ovary were located in the same quadrant with the results of antioxidant tests (DPPH, ABTS, and FRAP), indicating the high antioxidant activity of these plant part extracts of *P. ostii*. Compounds P3 (gallic acid), T2 (PGG), F1 (kaempferol), and F2 (quercitrin) also demonstrated the nearest distance with the antioxidant activity of the samples in the biplot. These results, combined with previous research, suggest that five compounds (gallic acid, PGG, *trans*-resveratrol, kaempferol, and quercitrin) are the predominant active compounds contributing to the antioxidant capacity in *P. ostii* (Yang et al., 2008; Chen and Chen, 2013; Shaikh et al., 2016; Pereira et al., 2018).

#### 3.6.3 CB1 and CB2 receptor agonist activity evaluation

**Figure 7:**
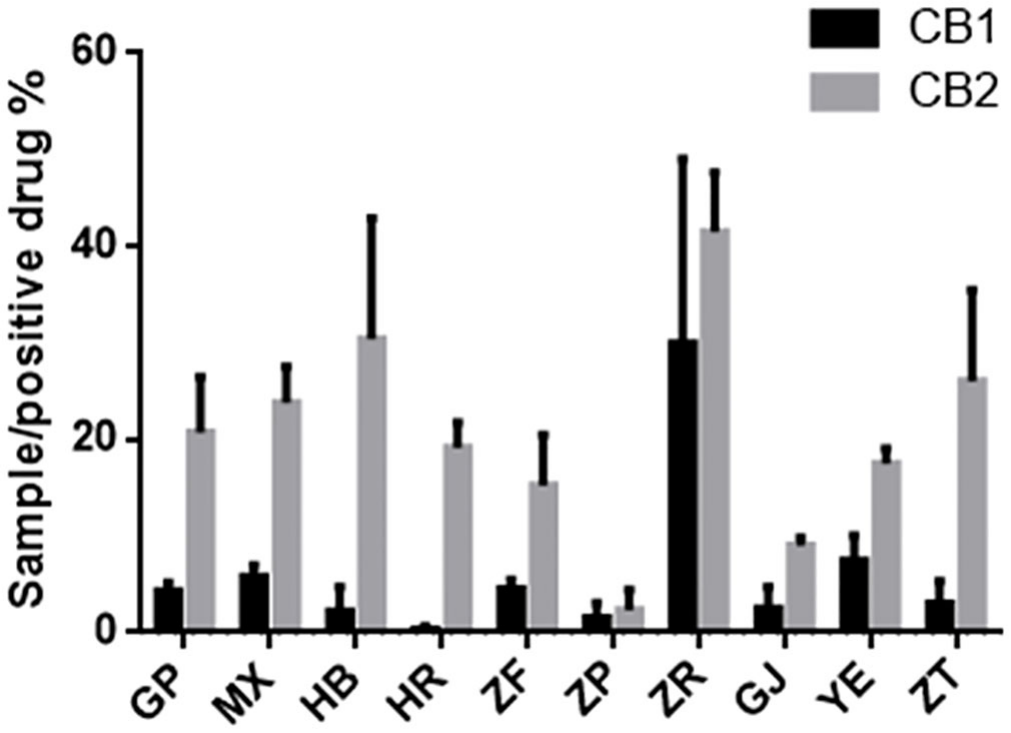
Agonist activity of plant extracts from 10 different parts of *Paeonia ostii* on cannabinoid receptors CB1 and CB2 (positive drug at 10^−6^ M concentration and samples at 10 μg/mL concentration).

The endogenous cannabinoid system plays a crucial role in various physiological functions, and the cannabinoid receptors CB1 and CB2 are members of the G protein family (Munro et al., 1993). Extensive research indicates that agonists of CB1 and CB2 receptors can exert anti-inflammatory, analgesic, microcirculation-improving, anti-cancer activities and treat neurodegenerative diseases (Mackie, 2006; Riether, 2012; Morales et al., 2016). Notably, the cannabinoid receptor-modulating drugs currently in use are mainly of plant origin (Newman and Cragg, 2007). To evaluate the agonist activity of different parts of *P. ostii* on CB1 and CB2 receptors, extracts from each plant part sample were tested, and the results are shown in **Figure 7**. It can be seen that at a concentration of 10 μg/mL, the agonist activity of all sample extraction on CB2 was significantly higher than that of CB1. Among the 10 different plant part extracts, the seed kernel extracts (i.e., the peony seed cakes) showed the strongest agonistic activity on CB1 and CB2. Specifically, they displayed approximately 28% and 41% of the activity of the positive drug (10^−6^ M) at a concentration of 10 μg/mL, while the other samples showed weak or negligible agonistic activity on CB1 activity (less than 10%). These findings suggest the potential of seed cakes as valuable medicinal resources. In the screening against CB2, in addition to the seed kernel extraction, extracts from other plant parts such as petals, root bark, root core, and branches were also active, further expanding the application potential of *P. ostii*.

## 4 Conclusions

*P. ostii* is an important medicinal and oil crop. Except for the root bark, seed kernels, and flowers, which are used for medicinal and edible purposes, most of the resources from other plant parts are discarded during production and processing. Considering the potential resource utilization value of these renewable botanical parts, this study reported the chemical properties of 10 plant parts of *P. ostii* by non-targeted metabolomics analysis based on UPLC-Q-TOF-MS technology and quantitative analysis of important biomarker components by UPLC-DAD. Furthermore, they were screened for their antioxidant and cannabinoid receptor agonist activities. Through this analysis, a total of 160 metabolites were identified from *P. ostii* through an in-house database. In the PCA scoring plot and HCA plot, root bark had obvious differences from other parts of *P. ostii* in chemical composition. In the PCA scoring chart, four categories can be observed. The root bark forms a separate cluster, showing obvious differences between the metabolic characteristics of the root bark and other parts. The metabolic profile of the root core is found to be more similar to that of the branches. Furthermore, the flowers (petals, stamens, and ovary) and leaves exhibit close proximity in the plot, suggesting a higher similarity in metabolic characteristics compared to other parts. Fifty-five compounds as the potential characteristic markers were identified in 10 different parts of *P. ostii* by S-Plot. Antioxidant activities assessed using ABTS, FRAP, and DPPH assays showed the greatest activity in leaves. Additionally, the discarded plant parts in production, such as root core, branches, stamens, and ovary, also demonstrated substantial antioxidant activities. The compounds gallic acid, PGG, *trans*-resveratrol, kaempferol, quercitrin, and monoterpene glycosides, most likely, are major contributors to the total antioxidant activities of the plant extracts of 10 different parts of *P. ostii*. In addition, *P. ostii* seed cakes (seed kernel extracts) of this study showed agonistic activity on CB1 and CB2 receptors, which can be used as a potential medicinal resource.

Herein, we focused on the moderately polar secondary metabolites in *P. ostii* and therefore used 70% methanol–water as a solvent to extract the plant samples. Different organs of the plant contain a wide variety of components, including small and large molecules, and low- and high-polarity compounds. Other types of components such as low polar (fatty acids and carotenoids) as well as large polar (polysaccharides, proteins, and amino acids), which were usually considered to be exploitable, should be included in further studies. By expanding the scope of analysis to encompass a broader range of compounds, a more comprehensive understanding of the chemical composition and potential uses of *P. ostii* can be achieved.

In addition to *P. ostii* as the source of oil peonies, another noteworthy species in the *Paeonia* Sect. Moutan, *Paeonia rockii* is extensively cultivated in the Loess Plateau of northwest China because of its unique biological characteristics. Like *P. ostii*, the comprehensive utilization of *P. rockii* plant resources also deserves attention (Bai et al., 2021). Additionally, recent studies have shown that extracts obtained from the flowers of *Paeonia* exhibit promising anti-photoaging effects, suggesting their potential application in the development of anti-photoaging skincare products (Lv et al., 2023). Following the theory of pharmaphylogeny, the phylogenetic relationship, chemical composition, and pharmacological activities of *Paeonia* spp. can be studied in the future to expand the application of existing medicinal plant resources, thus reasonably promoting the rational utilization of traditional Chinese medicine resources. Not only that, the whole *Paeonia* is rich in plant resources in China (Hong, 2021), and the potential plants that can be exploited include widely cultivated medicinal *P. lactiflora* and *P. veitchii* according to the theory of pharmacophylogeny (Gong et al., 2022). Therefore, the research ideas and results of this paper can provide a valuable reference for other plants of *Paeonia* to resource comprehensive utilization.

## Data availability statement

The original contributions presented in the study are included in the article/**Supplementary Material**. Further inquiries can be directed to the corresponding author.

## Author contributions

YZ and PL wrote the manuscript. CH and PX systemically revised the manuscript for important content. YZ and JS helped to complete the data analysis. YZ, PL, and KY completed the figures and tables. XW, YW, and YY helped with activity assays. CH proposed the concept. YZ and PL designed the structure of the manuscript. All authors read and approved the final manuscript.

## References

[B1] Bai Z. Ni J. Tang J. Sun D. Yan Z. Zhang J.. (2021). Bioactive components, antioxidant and antimicrob ial activities of Paeonia rockii fruit during development. Food Chem. 343, 128444. doi: 10.1016/j.foodchem.2020.128444 33131958

[B2] Bao Y. Qu Y. Li J. Li Y. Ren X. Maffucci K. G.. (2018). *In vitro* and in *vivo* antioxidant activities of the flowers and leaves from paeonia rockii and identification of their antioxidant constituents by UHPLC-ESI-HRMSn via pre-column DPPH reaction. Molecules 23 (2), 392. doi: 10.3390/molecules23020392 29439520 PMC6017382

[B3] Bi W. Shen J. Gao Y. He C. Peng Y. Xiao P. (2016). Ku-jin tea (Acer tataricum subsp. ginnala or A. tataricum subsp. theiferum), an underestimated functional beverage rich in antioxidant phenolics. J. Funct. Foods 24, 75–84. doi: 10.1016/j.jff.2016.04.002

[B4] Bothon F. T. D. Debiton E. Avlessi F. Forestier C. Teulade J.-C. Sohounhloue D. K. C. (2013). *In vitro* biological effects of two anti-diabetic medicinal plants used in Benin as folk medicine. BMC Complement. Altern. Med. 13, 51. doi: 10.1186/1472-6882-13-51 23452899 PMC3599091

[B5] Chen A. Y. Chen Y. C. (2013). A review of the dietary flavonoid, kaempferol on human health and cancer chemoprevention. Food Chem. 138 (4), 2099–2107. doi: 10.1016/j.foodchem.2012.11.139 23497863 PMC3601579

[B6] Dienaitė L. Pukalskienė M. Pukalskas A. Pereira C. V. Matias A. A. Venskutonis P. R. (2019). Isolation of strong antioxidants from paeonia officinalis roots and leaves and evaluation of their bioactivities. Antioxidants 8 (8), 249. doi: 10.3390/antiox8080249 31357649 PMC6721766

[B7] Gao L. Yang H. Liu H. Yang J. Hu Y. (2016). Extensive transcriptome changes underlying the flower color intensity variation in Paeonia ostii. Front. Plant Sci. 6. doi: 10.3389/fpls.2015.01205 PMC4702479 26779235

[B8] Gong X. Yang M. He C. Bi Y. Zhang C. Li M.. (2022). Plant pharmacophylogeny: review and future directions. Chin. J. Integr. Med. 28 (6), 567–574. doi: 10.1007/s11655-020-3270-9 33170942

[B9] Gu R. Rybalov L. Negrin A. Morcol T. Long W. Myers A. K.. (2019). Metabolic profiling of different parts of Acer truncatum from the Mongolian plateau using UPLC-QTOF-MS with comparative bioactivity assays. J. Agric. Food Chem. 67 (5), 1585–1597. doi: 10.1021/acs.jafc.8b04035 30675777

[B10] He C Bi W Shen J Peng Y Xiao P. (2016). Determination of ten stilbenes and their antioxidant activity of peony seed coat, seed kernel and seed coat extracts. China Journal Chinese Materia Medica. 41 (6), 1081–1086. doi: 10.4268/cjcmm20160618 28875674

[B11] Hong D. (2010). Peonies of the World: Taxonomy and Phytogeography (Scotland Edinburgh: Royal Botanic Garden, Kew).

[B12] Hong D. (2011). Peonies of the World: Polymorphism and Diversity (Scotland Edinburgh: Royal Botanic Garden, Kew).

[B13] Hong D. (2021). Peonies of the World: Phylgeny and Evolution (Scotland Edinburgh: Royal Botanic Garden, Kew).

[B14] Karolina T. Aneta W. Igor P. T. Paulina N. (2021). Anti-diabetic, anti-cholinesterase, and antioxidant potential, chemical composition and sensory evaluation of novel sea buckthorn-based smoothies. Food Chem. 338, 128105. doi: 10.1016/j.foodchem.2020.128105 33092003

[B15] Kolak U. Oztürk M. Ozgökçe F. Ulubelen A. (2006). Norditerpene alkaloids from Delphinium linearilobum and antioxidant activity. Phytochemistry 67 (19), 2170–2175. doi: 10.1016/j.phytochem.2006.06.006 16860354

[B16] Li Y. Bao Y. He H. Zhang N. (2023). Activity and mechanism of palbinone against hepatic fibrosis and inflammation. Acta Pharm. Sinica. 58 (2), 371–376. doi: 10.16438/j.0513-4870.2022-1050

[B17] Li P. Shen J. Wang Z. Liu S. Liu Q. Li Y.. (2021). Genus Paeonia: A comprehensive review on traditional uses, phytochemistry, pharmacological activities, clinical application, and toxicology. J. Ethnopharmacol. 269, 113708. doi: 10.1016/j.jep.2020.113708 33346027

[B18] Li S. Yuan R. Chen L. Wang L. Hao X. Wang L.. (2015). Systematic qualitative and quantitative assessment of fatty acids in the seeds of 60 tree peony (Paeonia section Moutan DC.) cultivars by GC-MS. Food Chem. 173, 133–140. doi: 10.1016/j.foodchem.2014.10.017 25466004

[B19] Liu S. Li Y. Yi F. Liu Q. Xiao P. (2020). Resveratrol oligomers from Paeonia suffruticosa protect mice against cognitive dysfunction by regulating cholinergic, antioxidant and anti-inflammatory pathways. J. Ethnopharmacol. 260, 112983. doi: 10.1016/j.jep.2020.112983 32442589

[B20] Liu P. Zhang Y. Gao J. Du M. Zhang K. Zhang J.. (2018). HPLC-DAD analysis of 15 monoterpene glycosides in oil peony seed cakes sourced from different cultivation areas in China. Ind. Crops Prod. 118, 259–270. doi: 10.1016/j.indcrop.2018.03.033

[B21] Liu P. Zhang L. Wang X. Gao J. Yi J. Deng R. (2019). Characterization of Paeonia ostii seed and oil sourced from different cultivation areas in China. Ind. Crops Prod. 133, 63–71. doi: 10.1016/j.indcrop.2019.01.054

[B22] Lv M. Yang Y. Choisy P. Xu T. Pays K. Zhang L.. (2023). Flavonoid components and anti-photoaging activity of flower extracts from six Paeonia cultivars. Ind. Crops Prod. 200, 116707. doi: 10.1016/j.indcrop.2023.116707

[B23] Mackie K. (2006). Cannabinoid receptors as therapeutic targets. Annu. Rev. Pharmacol. Toxicol. 46, 101–122. doi: 10.1146/annurev.pharmtox.46.120604.141254 16402900

[B24] Mao Y. Han J. Tian F. Tang X. Hu Y. Guan Y. (2017). Chemical composition analysis, sensory, and feasibility study of tree peony seed. J. Food Sci. 82 (2), 553–561. doi: 10.1111/1750-3841.13593 28135396

[B25] Morales P. Hernandez-folgado L. Goya P. Jagerovic N. (2016). Cannabinoid receptor 2 ( CB 2 ) agonists and antagonists: a patent update. Expert Opin. Ther. Patents 26 (7), 843–856. doi: 10.1080/13543776.2016.1193157 27215781

[B26] Munro S. Thomas K. L. Abu-Shaar M. (1993). Molecular characterization of a peripheral receptor for cannabinoids. Nature 365, 61–65. doi: 10.1038/365061a0 7689702

[B27] Newman D. J. Cragg G. M. (2007). Natural products as sources of new drugs over the last 25 years. J. Natural Prod. 70 (3), 461–477. doi: 10.1021/np068054v 17309302

[B28] Pan Y. Gao Z. Huang X.-Y. Chen J.-J. Geng C.-A. (2020). Chemical and biological comparison of different parts of Paeonia suffruticosa (Mudan) based on LCMS-IT-TOF and multi-evaluation in *vitro* . Ind. Crops Prod. 144 (132), 112028. doi: 10.1016/j.indcrop.2019.112028

[B29] Parker S. May B. Zhang C. Zhang A. Lu C. Xue C. (2016). A Pharmacological Review of Bioactive Constituents of Paeonia lactiflora Pallas and Paeonia veitchii Lynch. Phytother. Res. 30 (9), 1445–1473. doi: 10.1002/ptr.5653 27279421

[B30] Pereira M. M. de Morais H. Dos Santos Silva E. Corso C. R. Adami E. R. Carlos R. M.. (2018). The antioxidant gallic acid induces anxiolytic-, but not antidepressant-like effect, in streptozotocin-induced diabetes. Metab. Brain Dis. 33 (5), 1573–1584. doi: 10.1007/s11011-018-0264-9 29934859

[B31] Riether D. (2012). Selective cannabinoid receptor 2 modulators: a patent review 2009 – present. Expert Opin. Ther. Patents 22 (5), 495–510. doi: 10.1517/13543776.2012.682570 22537079

[B32] Rocchetti G. Senizza B. Zengin G. Okur M. A. Montesano D. Yildiztugay E. (2019). Chemical profiling and biological properties of extracts from different parts of Colchicum szovitsii subsp. Szovitsii. Antioxidants 8 (12), 632. doi: 10.3390/antiox8120632 31835669 PMC6943543

[B33] Ruixue D. Xiao Y. Chunxiao Q. Ye P. Jianglei Z. Pu L. (2019). Chemical compositions in different parts of the seeds of three Paeonia species. Food Sci. 40 (8), 141–148. doi: 10.7506/spkx1002-6630-20180429-375

[B34] Shaikh Q. U. A. Yang M. Memon K. H. Lateef M. Na D. Wan S.. (2016). 1,2,3,4,6-Pentakis[-O-(3,4,5-trihydroxybenzoyl)]-α,β-D-glucopyranose (PGG) analogs: design, synthesis, anti-tumor and anti-oxidant activities. Carbohydr. Res. 430, 72–81. doi: 10.1016/j.carres.2016.04.021 27196315

[B35] Shen J. Zhou Q. Li P. Wang Z. Liu S. He C.. (2017). Update on phytochemistry and pharmacology of naturally occurring Resveratrol oligomers. Molecules 22 (2050), 1–26. doi: 10.3390/molecules22122050 PMC6149893 29186764

[B36] Sindhi V. Gupta V. Sharma K. Bhatnagar S. Kumari R. Dhaka N. (2013). Potential applications of antioxidants – A review. J. Pharm. Res. 7 (9), 828–835. doi: 10.1016/j.jopr.2013.10.001

[B37] Sumner L. W. Amberg A. Barrett D. Beale M. H. Beger R. Daykin C. A.. (2007). Proposed minimum reporting standards for chemical analysis Chemical Analysis Working Group (CAWG) Metabolomics Standards Initiative (MSI). Metabolomics 3 (3), 211–221. doi: 10.1007/s11306-007-0082-2 24039616 PMC3772505

[B38] Tian X. Guo S. Zhang S. Li P. Wang T. Ho C.. (2020). Chemical characterization of main bioactive constituents in *Paeonia ostii* seed meal and GC-MS analysis of seed oil. J. Food Biochem. 44 (1), 1–12. doi: 10.1111/jfbc.13088 31646682

[B39] Vinaixa M. Schymanski E. L. Neumann S. Navarro M. Salek R. M. Yanes O. (2016). Mass spectral databases for LC/MS- and GC/MS-based metabolomics: State of the field and future prospects. TrAC Trends Ana. Chem. 78, 23–35. doi: 10.1016/j.trac.2015.09.005

[B40] Wang Z. Chen Y. Liu S. Li H. Li W. Dong L.. (2018). Morphological, microscopic, multiple-component assay and fingerprinting based systematic research on quality evaluation of Moutan Cortex (Paeonia suffruticosa). China J. Chin. Mater. Medica 43 (13), 296–305. doi: 10.19540/j.cnki.cjcmm.20180510.002 30111048

[B41] Wang L. Fumio H. Aya S. Aoki N. Jiajue L. Sakata Y. (2004). Chemical taxonomy of the Xibei tree peony from China by floral pigmentation. J. Plant Res. 117, 47–55. doi: 10.1007/s10265-003-0130-6 14685820

[B42] Wang Z. He C. Peng Y. Chen F. Xiao P. (2017a). Origins, phytochemistry, pharmacology, analytical methods and safety of Cortex moutan (Paeonia suffruticosa Andrew): a systematic review. Molecules 22 (6), 946. doi: 10.3390/molecules22060946 28590441 PMC6152737

[B43] Wang Z. Shen J. Li P. Liu S. Yi F. Liu H.. (2017b). Research on quality markers of Moutan cortex: quality evaluation and quality standards of Moutan cortex. Chin. Herbal Medi. 9 (4), 307–320. doi: 10.1016/S1674-6384(17)60110-2

[B44] Wang Z. Zhu C. Liu S. He C. Chen F. Xiao P. (2019). Comprehensive metabolic profile analysis of the root bark of different species of tree peonies (Paeonia Sect. Moutan). Phytochemistry 163, 118–125. doi: 10.1016/j.phytochem.2019.04.005 31048131

[B45] Yang J. Guo J. Yuan J. (2008). *In vitro* antioxidant properties of rutin. LWT Food Sci. Technol. 41 (6), 1060–1066. doi: 10.1016/j.lwt.2007.06.010

[B46] Yang D. Li L. Xue S. Fang X. (2017). Research progress on comprehensive utilization of oil peony. Hubei Agric. Sci. 56 (21), 4096–4100. doi: 10.14088/j.cnki.issn0439-8114.2017.21.025

[B47] Yang Y. Yang F. Xiong Y. (2011). Comprehensive utilization and development prospect of peony flowers. Northern Horticult. 01, 67–69.

[B48] Yoo T.-K. Jeong W. T. Kim J. G. Ji H. S. Ahn M. A. Chung J. W.. (2021). UPLC-ESI-Q-TOF-MS-based metabolite profiling, antioxidant and anti-inflammatory properties of different organ extracts of Abeliophyllum distichum. Antioxidants 10 (1), 70. doi: 10.3390/antiox10010070 33430473 PMC7827262

[B49] Zhang H. Li X. Wu K. Wang M. Liu P. Wang X.. (2017). Antioxidant activities and chemical constituents of flavonoids from the flower of Paeonia ostii. Molecules 22 (1), 5. doi: 10.3390/molecules22010005 PMC6155618 28025554

[B50] Zhang Y. Liu P. Gao J. Wang X. Yan M. Xue N.. (2018). Paeonia veitchii seeds as a promising high potential by-product: Proximate composition, phytochemical components, bioactivity evaluation and potential applications. Ind. Crops Prod. 125, 248–260. doi: 10.1016/j.indcrop.2018.08.067

[B51] Zhang X. Shi Q. Ji D. Niu L. Zhang Y. (2017). Determination of the phenolic content, profile, and antioxidant activity of seeds from nine tree peony (Paeonia section Moutan DC.) species native to China. Food Res. Int. 97, 141–148. doi: 10.1016/j.foodres.2017.03.018 28578034

[B52] Zhang X. Zhai Y. Yuan J. Hu Y. (2019). New insights into Paeoniaceae used as medicinal plants in China. Sci. Rep. 9 (1), 1–10. doi: 10.1038/s41598-019-54863-y 31804561 PMC6895042

[B53] Zhao M. Wu S. P. (2019). A review of the ethnobotany, phytochemistry and pharmacology of tree peony (Sect. Moutan). South Afr. J. Botany 124, 556–563. doi: 10.1016/j.sajb.2019.05.018

[B54] Zhou T. Liu Y. Zhang L. (2020). Progress and prospects of research on new resources peony seed oil. J. Heze Coll. 42 (02), 100–103. doi: 10.16393/j.cnki.37-1436/z.2020.02.021

