# Supplementary material for: Comprehensive comparison of different parts of Paeonia ostii, a food-medicine plant, based on untargeted metabolomics, quantitative analysis, and bioactivity analysis
Source: Front Plant Sci. 2023 Aug 29;14:1243724. doi: 10.3389/fpls.2023.1243724 (PMC10497777; doi:10.3389/fpls.2023.1243724)
Supplement: Supplementary file 1 [file DataSheet_1.docx]

Supplementary Material

**Comprehensive comparison of different parts of *Paeonia ostii*, a food-medicine plant: based on untargeted metabolomics，quantitative analysis and bioactivity analysis**

**Yaping Zheng^1^, Pei Li^1^, Jie Shen^1,2^, Kailing Yang^1^, Xinyan Wu^1^ Yue Wang^1^, Yuhe Yuan^3^, Peigen Xiao^1^, Chunnian He^1*^**

*** Correspondence:** Corresponding Author: cnhe@implad.ac.cn

# Supplementary Figures and Tables

## Supplementary Figures

**Root core**

##

**Root bark**

**Stamens**

**Petals**

**Seed kernels**

**Seed coats**

**Ovary**

**Fruit shells**

**Supplementary Figure 1.** Base peak chromatogram (BPI) of each plant part extract of *P. ostii*


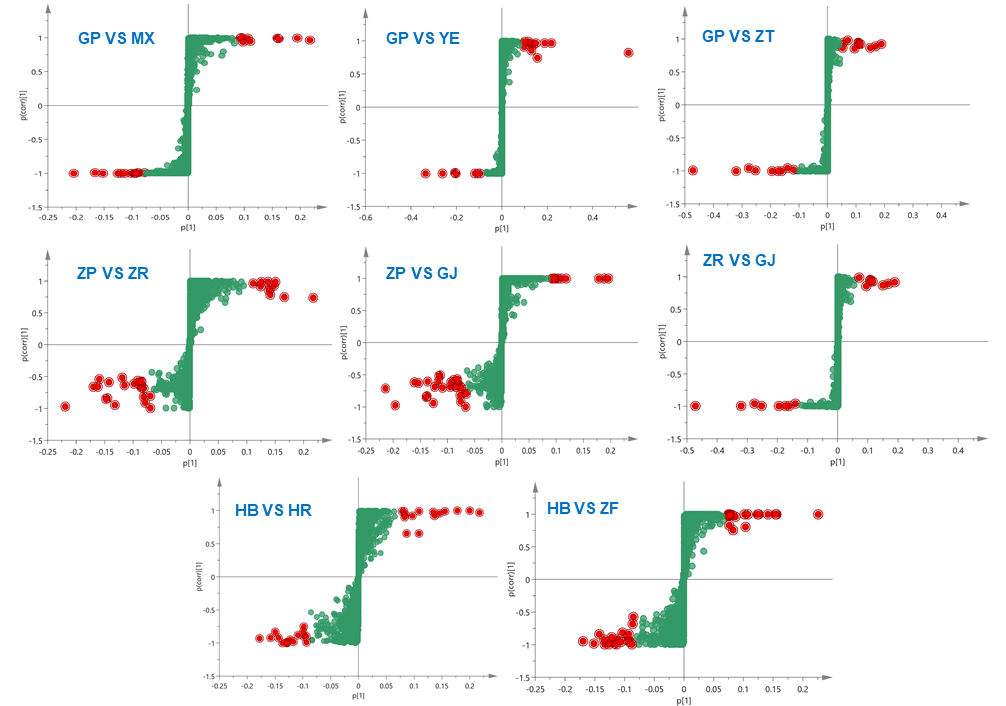


**Supplementary Figure 2.** S-plot score from the eight different comparison models along with selected candidate marker compounds from each model. Please refer to Table 2 for the reference of the groups. (GP=root bark, MX=root core, ZT=branches, YE=leaves, HB=petals, HR=stamens, ZF=ovary, ZP=seed coats, ZR=seed kernels; GJ=fruit shells)

- 1. **Supplementary Tables**

**Supplementary table 1.** The plant samples of ten different parts of *P. ostii*

| **Plant parts** | **Abbreviations** | **Collecting time** | **Specimen code** |
| --- | --- | --- | --- |
| leaves | YE | 2017-5-9 | FH2 |
| petals | HB | 2017-5-9 | FB2 |
| stamens | HR | 2017-5-9 | FC1 |
| ovary | ZF | 2017-5-9 | YD1 |
| seed coat | ZP | 2017-9-12 | FE3 |
| seed kernels | ZR | 2017-9-12 | FF2 |
| fruit shells | GJ | 2017-9-12 | FG1 |
| branches | ZT | 2017-9-12 | FI1 |
| root bark | GP | 2016-10-22 | YE1 |
| root core | MX | 2016-10-22 | FA3 |

**Supplementary table 2.** All compounds identified from ten *P. ostii* based on Progenesis QI software

**Supplementary table 3.** Statistical parameters obtained from different OPLS-DA models according to UPLC-QTOF-MS data and the number of marker compounds selected in S-Plot

| Groups | Scaling | Components | R^2^ | Q^2^ | Markers (-1) | Markers (1) |
| --- | --- | --- | --- | --- | --- | --- |
| GP(-1)VS MX(1) | Pareto | 2 | 98 | 99.6 | 14 | 12 |
| GP(-1)VS YE(1) | Pareto | 2 | 99.5 | 99.9 | 7 | 12 |
| GP(-1)VS ZT(1) | Pareto | 2 | 95.2 | 99.5 | 9 | 12 |
| HB(-1)VS HR(1) | Pareto | 3 | 91.1 | 98.8 | 18 | 15 |
| HB(-1)VS ZF(1) | Pareto | 2 | 90.4 | 99.5 | 20 | 19 |
| ZP(-1)VS ZR(1) | Pareto | 2 | 93.9 | 99.4 | 23 | 19 |
| ZP(-1)VS GJ(1) | Pareto | 2 | 97.2 | 99.8 | 33 | 12 |
| ZR(-1)VS GJ(1) | Pareto | 2 | 99.7 | 99.9 | 13 | 12 |
